# Cheetahs (*Acinonyx jubatus*) running the gauntlet: an evaluation of translocations into free-range environments in Namibia

**DOI:** 10.7717/peerj.1346

**Published:** 2015-10-22

**Authors:** Florian J. Weise, Joseph R. Lemeris, Stuart J. Munro, Andrew Bowden, Cicelia Venter, Marlice van Vuuren, Rudie J. van Vuuren

**Affiliations:** 1 Research, N/a’an ku se Research Programme, Windhoek, Namibia; 2 School of Science and the Environment, Division of Biology and Conservation Ecology, The Manchester Metropolitan University, Manchester, United Kingdom; 3 Big Cats Initiative, National Geographic Society, Washington District of Columbia, United States of America; 4 Directorate, N/a’an ku se Foundation, Windhoek, Namibia

**Keywords:** *Acinonyx jubatus*, Cheetah, Relocation, Namibia, Conflict, Management

## Abstract

Following dramatic range and population declines, the cheetah is Africa’s most endangered large felid. In Namibia, private land managers still trap cheetahs but increasingly consider moving animals instead of killing them. Across Africa, managers have translocated perceived conflict carnivores for decades, but rarely evaluated their actions. We analyse the outcomes of 15 cheetah translocations (for 23 adults and 10 dependent offspring) into free-range environments in Namibia. We released cheetahs at an average distance of 419.6 km ± 216.1 km SD (range: 71–816 km) after captive periods ranging from 1–1,184 days (350.6 days ± 439.0 days SD). An individual’s ability to survive the first year predominantly determined the overall translocation success of 40%. Post-release conflict and homing had less impact on success. Cheetah survival was lowest in the first three months after release. Human persecution (50% of deaths) and spotted hyaenas (29% of deaths) had the highest effect on survival. The degree of habituation to humans acquired during captivity significantly influenced chances of survival. Cheetahs surviving the initial post-release period (∼90 days) often settled into ranges and females reproduced successfully. However, all individuals exhibited extensive movements, frequently roaming >4,000 km^2^ in the first six months after release (with a maximum of 19,743 km^2^ in 112 days), resulting in low release site fidelity. Soft release and larger recipient area size did not improve site fidelity. Based on these outcomes, we evaluated which unfenced conservation areas in Namibia could potentially receive cheetahs. We found that there are currently few public and/or private reserves large enough to contain the movement profiles we observed in this study. This suggests that most translocations will result in cheetahs re-entering farmlands where they face a high risk of persecution. In conclusion, translocations into unconfined areas can successfully conserve individual cheetahs. Due to high mortality and unpredictable outcomes, however, conservation efforts need to focus on improving tolerance of cheetahs in commercial livestock and game farming areas in order to reduce the number of indiscriminately trapped animals.

## Introduction

The cheetah is Africa’s most endangered large felid and classified as Vulnerable by the IUCN (Durant et al., 2008). On private farms in Namibia—currently home to the single largest population in any country (Marker, 2005; Purchase et al., 2007)—cheetahs interfere with human interests by killing livestock and valuable game (Marker et al., 2003c; Morsbach, 1987; Voigt et al., 2014). As a consequence of perceived and actual conflict, annual cheetah removals from commercial ranches historically ranged between 650–890 individuals (Morsbach, 1987), resulting in a reported loss of 9,588 cheetahs through lethal actions (conflict control and trophy hunting) or export between 1978 and 1994 (Nowell, 1996). More recently (2008–2014), we gathered information of lethal removal of 267 cheetahs across 26,090 km^2^ of commercially managed farms in Namibia. The sample (221 properties) represents ∼8% of Namibia’s free-hold farming landscape (Mendelsohn, 2006) and our observations translate into an average annual persecution of 0.51 cheetahs/100 km^2^ on these private lands (*cf*. 0.5 cheetahs/farm (*n* = 126 farms); Gaerdes, 1974). While most managers do not persecute cheetahs continuously, indiscriminate lethal control can be as high as 82 cheetahs on a single property in two years (F Weise, pers. obs., 2013) and total over 200 individuals across a farmer’s lifetime (Lindsey et al., 2013a).

It is therefore not surprising that translocation of perceived conflict cheetahs has been a common strategy in Namibia (du Preez, 1970; Marker et al., 2003b; T Hoth, pers. comm., 2014), as well as in other areas of southern Africa (Marnewick et al., 2009; Phiri, 1996; Purchase, Vhurumuku & Purchase, 2006). In some countries, e.g. Botswana, translocation continues to be employed as a standard response to livestock conflicts involving cheetahs, but rarely with sufficient post-release follow-up (Boast, Good & Klein, 2015). In Namibia, 49% of land managers (*n* = 221) considered translocation of conflict carnivores as a non-lethal management option. In the sampled areas, 60% of managers believed that cheetahs killed livestock and/or valuable game and 39 managers translocated cheetahs in the past, with either the state wildlife department or a non-governmental organisation (F Weise, 2008–2014, unpublished data).

The evaluation of conflict predator translocations in southern Africa is receiving increasing attention, in part reflecting advances in tracking technology that enable detailed assessments. Success criteria typically include a combination of survival (Marnewick et al., 2009), post-release movements and conflict resolution (Stander, 1990a; Stander et al., 1997; Weilenmann et al., 2010). Some studies also determined translocation costs (Boast, Good & Klein, 2015; Weise, Stratford & van Vuuren, 2014) and the availability of suitable recipient habitat for such events (Weise et al., 2015). Despite many potential caveats (see review by Linnell et al., 1997), carnivore translocations continue to be in high demand among land managers (also see Boast, Good & Klein, 2015). Therefore, it is important that evaluations incorporate successes and failures alike to prevent bias in future management decisions (Linnell et al., 1997; Massei et al., 2010).

Here, we provide the first detailed evaluation of cheetah translocations carried out into private free-range conservation areas in Namibia. Case-specific translocation objectives varied, reflecting attempts to rehabilitate orphans or to mitigate conflict. Standard translocation objectives included: (i) to return cheetahs into free-range environments with minimal potential for post-release conflict; (ii) to enable their contribution to the wild gene pool; (iii) to alleviate conflict at source sites; and (iv) to determine the efficacy of cheetah translocations into unrestricted environments. We assess translocations in light of their biological results; including survivorship, movements, reproduction, and prey. Furthermore, we consider pre- and post-release conflict, translocation distance, release mode, and length of temporary captivity as important variables. Finally, we use our results, and those from other cheetah translocation studies in southern Africa, to determine suitable recipient areas across Namibia’s protected area (PA) network.

## Methods

### Subjects and success definition

**Figure 1:**
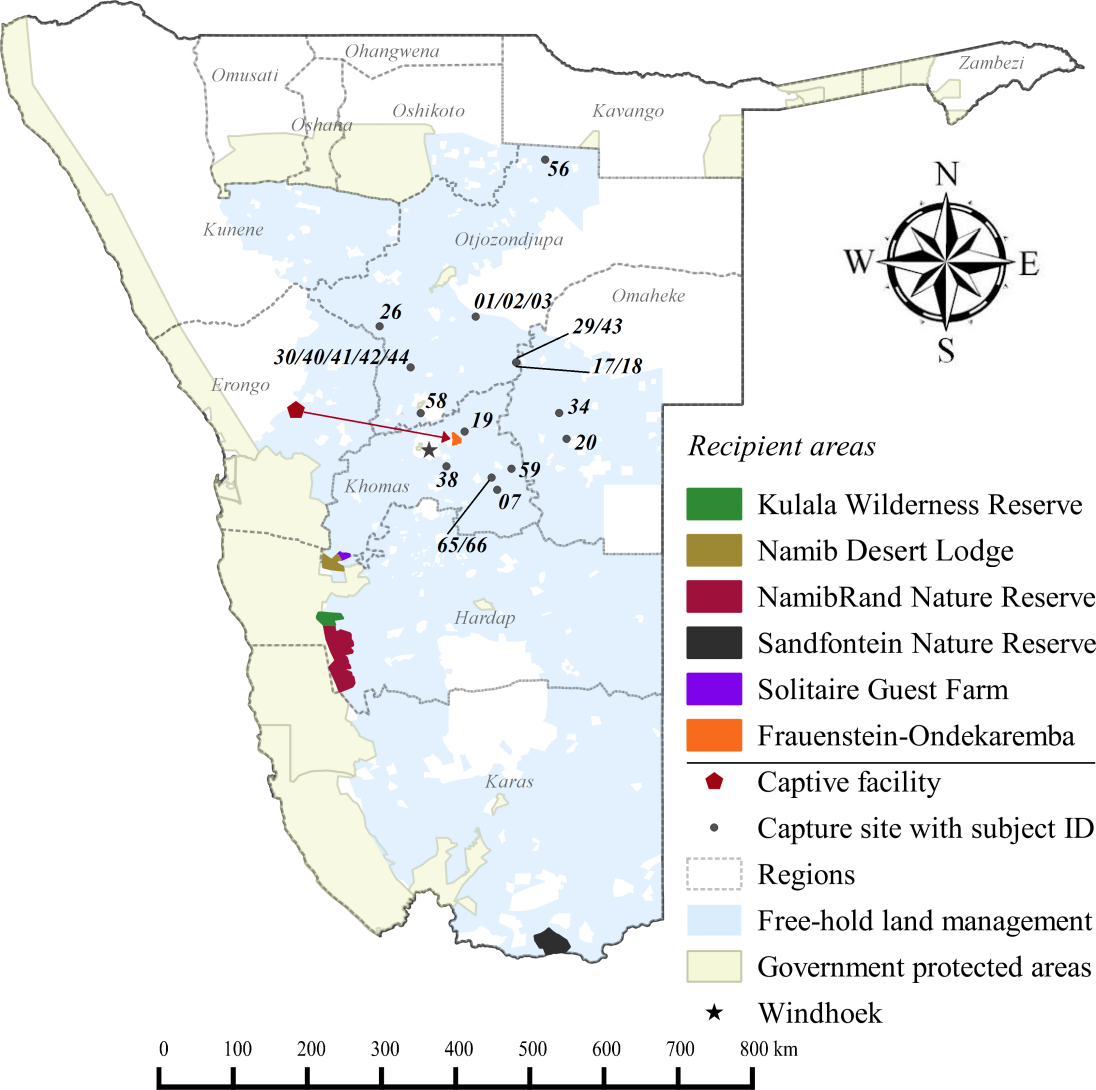
Cheetah capture sites and recipient areas in Namibia. Sites shown in relation to free-hold farmlands and government protected areas.

Free-hold land managers trapped study cheetahs on Namibia’s commercial farmlands between May 2008 and December 2012 (Fig. 1). They reported animals to the state wildlife department and requested translocation as an alternative to lethal control. In the present cases, government were unable to attend to trapped individuals at short notice and the animals were moved into a registered captive facility (Fig. 1) pending further decision on their management (i.e., permanent captivity vs. release). Contrary to a relocation programme in South Africa (Buk & Marnewick, 2010), Namibian managers did not receive financial rewards for translocating cheetahs instead of killing them.

Based on their specific background and the supporting evidence available, we classified cheetahs as livestock raiders (observed at kills and captured within 24 h of the incident), indiscriminate captures (those suspected of killing expensive game species and cases without evidence of livestock predation involvement), or orphans (trapped at pre-dispersal age without mothers) (Table 1).

**Table 1:** Subject details of translocated cheetahs in Namibia, 2008–2012.

ID	Sex	Estimated age (years)	Weight (kg)	Capture region— release area	Year^a^	Capture reason—background	Captivity (days)	Translocation distance (km)	Release mode (acclimation)— social composition	Degree of habituation	Transmitter type
Aju01	F	2–3	37	Otjozondjupa–NRNR	2008	Indiscriminate	10	526	Hard–sibling group	Wild	VHF^b^
Aju02	F	2–3	33	Otjozondjupa–NRNR	2008	Indiscriminate	10	526	Hard–sibling group	Wild	ID
Aju03	M	2–3	38	Otjozondjupa–NRNR	2008	Indiscriminate	10	526	Hard–sibling group	Wild	VHF^b^
Aju07	F	7–9	33	Khomas–NRNR	2008	Livestock raider	61	331	Hard–mother with 2 offspring	Wild	VHF^b^
Aju17	M	5–7	44	Otjozondjupa–NRNR	2009	Livestock raider	175	490	Hard–individual	Wild	GPS^c^ ARGOS
Aju18	F	5–7	43	Otjozondjupa–NRNR	2009	Livestock raider	157	470	Hard–individual	Wild	GPS^c^ ARGOS
Aju19	M	3–5	49	Khomas–KWR	2009	Indiscriminate	62	312	Hard–artificial group	Wild	VHF^d^
Aju20	F	6–8	37	Omaheke–KWR	2009	Indiscriminate	37	396	Hard–artificial group	Wild	GPS^c^ ARGOS
Aju26	M	2–3	44	Otjozondjupa–FOC	2009	Indiscriminate	12	182	Hard–individual	Wild	GPS^d^ cell
Aju29	F	3–4	38	Otjozondjupa–NRNR	2010	Orphan–rehab	596	482	Soft (10 weeks)–artificial group	Semi-habituated	VHF^d^
Aju30	M	3–4	41	Otjozondjupa–NRNR	2010	Orphan–rehab	446	431	Soft (10 weeks)–artificial group	Semi-habituated	GPS^c^ ARGOS
Aju34	M	3–4	43	Omaheke–FOC	2010	Indiscriminate	47	137	Hard–individual	Wild	VHF^d^
Aju38	M	3–4	37	Khomas–SGF	2011	Indiscriminate	153	259	Soft (18 weeks)–individual	Wild	GPS^c^ ARGOS
Aju40	F	3–4	37	Otjozondjupa–NDL	2012	Indiscriminate	1,184	289	Soft (38 weeks)–sibling group	Habituated	GPS^c^ ARGOS
Aju41	F	3–4	38	Otjozondjupa–NDL	2012	Indiscriminate	1,184	289	Soft (38 weeks)–sibling group	Habituated	VHF^d^; GPS^c^
Aju42	M	3–4	29	Otjozondjupa–SNGR	2011	Orphan–rehab	1,055	816	Soft (18.5 weeks)–artificial group	Habituated	VHF^b^
Aju43	M	3–4	32	Otjozondjupa–SNGR	2011	Orphan–rehab	1,106	806	Soft (18.5 weeks)–artificial group	Habituated	GPS^c^ ARGOS
Aju44	M	3–4	32	Otjozondjupa–SNGR	2011	Orphan–rehab	1,055	816	Soft (18.5 weeks)–artificial group	Habituated	VHF^d^
Aju56	F	5–7	45	Otjozondjupa–FOC	2012	Indiscriminate	169	402	Hard–mother with 3 offspring	Wild	GPS^c^ satellite
Aju58	F	5–7	40	Otjozondjupa - NRNR	2012	Indiscriminate	260	372	Soft (4 weeks)–mother with 2 offspring	Semi-habituated	GPS^d^ satellite
Aju59	F	4–6	38	Khomas–SNGR	2012	Indiscriminate	272	659	Soft (1 week)–mother with 3 offspring	Semi-habituated	GPS^d^ satellite
Aju65	M	6–7	51	Khomas–FOC	2012	Indiscriminate	1	71	Hard–male coalition	Wild	VHF^d^
Aju66	M	6–7	53	Khomas–FOC	2012	Indiscriminate	2	71	Hard–male coalition	Wild	GPS^d^ satellite

**Notes.**

a Year of release.

b Advanced Telemetry Systems, Insanti, USA.

c Sirtrack, Hawkes Bay, NZ.

d Africa Wildlife Tracking, Pretoria, RSA.

NRNR NamibRand Nature Reserve KWR Kulala Wilderness Reserve FOC Frauenstein–Ondekaremba Complex NDL Namib Desert Lodge SGF Solitaire Guest Farm SNGR Sandfontein Private Nature and Game Reserve (see Fig. 1)

We define success to include survival for at least one year, no homing to the original capture site or captive facility, and minimal livestock conflict (≤5 livestock per year) that was considered agreeable for compensation. However, landowners received compensation for any verifiable damage from translocated cheetahs. Reproduction—the ultimate indication of biological success—was not a condition for success, but it remained a key criterion in success evaluations. Although generally desirable (e.g., Boast, Good & Klein, 2015), site fidelity was also not a prerequisite for translocation success because all subjects were released into environments permitting free choice of movement.

### Management of cheetahs

Supplemental Information 1 provides a detailed description of captivity conditions, immobilisation protocols, and transportation. When we obtained immediate permission for translocation, cheetahs were only admitted to the captive facility to carry out medical examination and for fitting of tracking technology, followed by transfer to their release site. We minimised captive times as much as was practicable. Prolonged captivity (Table 1) resulted from delays in sourcing of recipient sites, rearing of orphans, presence of juvenile offspring, and issuing of permits. We kept study cheetahs with minimal human contact to avoid loss of natural fear. We assessed the degree of habituation to human presence and vehicles (Table 1) using discrete behavioural responses as classification criteria (Supplemental Information 2).

Licensed veterinary personnel (registered with the Namibian Veterinary Council) immobilised cheetahs to enable health assessments, fitting of tracking technology, recording of body measurements and collection of biological samples (Supplemental Information 1). We marked cheetahs with ID-microchips (subcutaneously) for permanent identification.

We define translocation distance as the linear distance from source site to release locality. We preferred long distances (>200 km) to prevent homing (Linnell et al., 1997; Marker, 2002), but distances were influenced by availability of recipient areas. We released cheetahs using hard releases, i.e., directly from the transport crate after being placed at a permanent water source for several hours, or through soft releases, i.e., following variable periods of acclimation in holding pens (Table 1) that measured 1–500 ha.

### Recipient areas and release considerations

Recipient areas (Supplemental Information 3) were privately owned nature reserves that promoted large carnivore population recovery. Translocated cheetahs contributed to ecosystem restoration efforts as well as non-consumptive wildlife tourism. Formal feasibility studies were not available and we selected release sites in correspondence with reserve management and the national wildlife authorities (B Beytell, pers. comm., 2008–2010). The reserves provided permanent water, adequate natural prey compositions, and a known local predator guild (Supplemental Information 3). All sites were located within extant species range, with estimated low densities of <2.0 cheetahs/100 km^2^ at the time that releases occurred (Hanssen & Stander, 2004; Stein et al., 2012).

None of the recipient areas had predator-proof fencing (Supplemental Information 3). Therefore, we awarded preference to reserves providing connectivity with large government PAs (Fig. 1). Because we could not ascertain site fidelity for releases into unconfined areas (Purchase, Vhurumuku & Purchase, 2006; Marker, 2002), we also employed a simple data sharing system to inform land managers of the movements of released cheetahs (Supplemental Information 1) and to enable recapture of individuals if necessary. We released cheetahs with a known livestock depredation background only into the largest reserve (i.e., NamibRand) with highest PA connectivity. Recipient reserves actively supported monitoring efforts and provided important logistic support, particularly in terms of cheetah husbandry in soft release enclosures. Reserves also informed surrounding land managers of translocation events and facilitated conflict mitigation through compensation agreements.

We implemented releases as staggered events to allow for sufficient settling and acclimation of previously translocated cheetahs. We maintained an inter-release interval of >4 months for subsequent releases into the same reserve. We released most cheetahs into areas where they experienced novel competition with spotted hyaena (*Crocuta crocuta*) but none of the recipient areas had free-ranging lions (*Panthera leo*). There was no supportive post-release management other than the recapture of one individual and the re-release of one accidentally trapped subject.

### Monitoring

Except for sub-adults released alongside their mothers and one female tagged with an ID collar as part of a group release, we fitted all subjects with tracking devices to enable intensive post-release monitoring. External transmitters included Very High Frequency (VHF) radio-collars as well as dual VHF-GPS (Global Positioning System) satellite transmission collars (Table 1). Battery life times were ∼36 months for VHF units and 12–30 months for GPS devices. Transmitter weight was <1.5% of an individual’s body mass. We fitted collars so that they allowed for expected neck growth of pre-prime individuals.

We collected positional data with standard VHF telemetry methods (Amlaner Jr & Macdonald, 1980; Kenward, 2001) and via remote GPS satellite telemetry. During direct observations, we took care not to disturb or displace animals. We attempted to locate VHF-tagged cheetahs at least once per week and telemetry efforts generally concentrated on these subjects. GPS units recorded and relayed 1–6 daily locations. Consequently, daily detection probability was <1.0 for VHF-tagged cheetahs and ∼1.0 for subjects with active GPS trackers.

We adjusted GPS sampling regimes to enable investigation of suspected kill sites and to assess conflict involvement of translocated cheetahs. Data sharing with land managers outside of PAs was an important monitoring element (protocol detail in Supplemental Information 1). If we could not locate a cheetah for several weeks, or GPS units failed early, we employed aerial telemetry from a Cessna 182 aircraft to ascertain the individual’s fate. When direct observations were not possible, we calculated locations with software LOCATE II (Nams, 1990) using triangulation of the strongest VHF signal bearings. Camera trap records and observation reports supplemented tracking information.

The Ministry of Environment and Tourism endorsed all research activities (permit numbers: 1254/2008; 1354/2009; 1459/2010; 1459/2011; 1782/2013; 1888/2014) and an ethics approval was obtained from Manchester Metropolitan University (clearance: RD1-06979434-20130923).

### Data analysis

We analysed data with software R v.3.1.0. (R Core Team, 2014) and JMP v.11.0 (SAS Institute, 2013). Spatial analyses were computed with ArcGIS v.10.1 (ESRI, 2013) and QGIS v.2.6-2.8 (QGIS Development Team, 2014). After removing outliers and low-quality GPS fixes, we standardised telemetry data to one location per day (closest to 12:00 GMT) (Mizutani & Jewell, 1998). Although this selection violates assumptions of auto-correlation between subsequent positions (Rooney, Wolfe & Kayden, 1998; Swihart & Slade, 1985), it reduces auto-correlation significantly (De Solla, Bonduriansky & Brooks, 1999) and provides a useful approach to enable comparisons between VHF- and GPS-tagged individuals and between GPS-collared individuals with variable sampling regimes.

To determine the duration of exploratory movements, we calculated 100% Minimum Convex Polygon (MCP) values for 10-day increments starting from the day of release. This 10-day period was progressively shifted by one day for the first year to assess when range values reached stable asymptotes (Weise et al., 2015). We confirmed asymptote stability by determining the date when percentage increase in total progressive 100% MCP range values was no longer significant (Gautestad & Mysterud, 1995). For animals with sufficient sample sizes, we calculated home ranges for periods excluding initial orientation and explorations. We measured home ranges as individually smoothed movement-based 50% and 95% kernel density estimations (following AUC-methods in Cumming & Cornélis, 2012) and 50% and 100% MCPs (Mohr, 1947) using the Spatial Analyst Movement Ecology Tools Extension v.10.2.2 (Wall, 2014).

We used percentage overlap of daily locations with the recipient area (i.e., release reserve) and other PAs (private and/or public conservation areas) as a measure of site fidelity for the first 12 months. We assessed homing behaviour using package Circular in R (Lund & Agostinelli, 2013) by calculating bearing angles and distances between an individual’s last known location and release location relative to their capture site. We adjusted bearing angles to set an individual’s ‘true home’ (i.e., original capture site) direction to 0°. We normalised all distances to a scale from zero to one, representing the distance between a cheetah’s capture and release sites. Cheetahs successfully homed if they moved the entire translocation distance towards the capture location, within 22.5°on either side of true home (Fies, Martin & Blank, 1987), or returned to the captive facility.

We assessed cheetah survival by calculating Kaplan–Meier survivorship estimates with the product limit estimator (Kaplan & Meier, 1958) using a staggered entry design (Pollock et al., 1989). This method allows for inclusion of animals entering the study at irregular intervals (Marnewick et al., 2009) and does not discriminate against individuals with unknown fate when collars fail or deplete. We report cheetah prey from direct, opportunistic observations of hunting events (with binoculars) and from carcasses investigated after detection of GPS clusters that indicated potential kills (Knopff et al., 2009). Therefore, prey results are limited to diurnal hunts and they exclude small prey species with low detectability.

### Potential recipient areas in Namibia

Considering cheetah ecology, the outcomes of this study, and those of other published cheetah translocations (Table 2), we modified the ArcGIS-based geo-processing tool CaTSuiT (Lemeris Jr, 2013) to determine cheetah recipient area suitability across conservation areas in Namibia—including national parks (137,451.5 km^2^), private reserves (4,883.9 km^2^), and gazetted communal conservancies (160,245.5 km^2^) (Supplemental Information 4). We defined recipient area suitability to include five conditions (Table 2) at equal parameter weighting. Hence, if an area did not meet all of these conditions, it was considered unsuitable. The rationale for this conservative approach was to maximise chances of post-release survival and reproduction, whilst minimising the risk of conflict and persecution (Table 2).

**Table 2:** Model input parameters used to determine cheetah recipient area suitability in Namibia.

Suitability condition	Exclusion criteria	Consideration [supporting studies]	Source of model input data
Land-use	Areas without designated protected status (e.g., commercial, communal farmlands)	(1) Reduce risk of conflict or persecution outside of designated recipient area (Boast, Good & Klein, 2015; du Preez, 1970; Houser et al., 2011; Marker et al., 2003b; Phiri, 1996)	(Mendelsohn et al., 2002; ConInfo, 2010; ConInfo, 2011)
Intra-guild competition	Medium and high lion occurrence Medium and high spotted hyaena occurrence	(1) Improve probability of post-release survival and successful reproduction (2) Reduce probability of kleptoparasitism (Caro, 1994; Eaton, 1974; Durant, 1998a; Durant, 2000; Hayward et al., 2006; Laurenson, 1994; Marnewick et al., 2009; Mills, Broomhall & du Toit, 2004; Purchase, Vhurumuku & Purchase, 2006; Stander, 1990b; Wachter et al., 2011)	Stein et al., 2012
Intra-specific competition	High occurrence of conspecifics No cheetah occurrence	(1) Provide connectivity and mating opportunities with free-ranging conspecifics at low–medium occurrence (2) Reduce risk of intra-specific competition/aggression (particularly for males) (3) Local carrying capacity (Caro, 1994; Caro & Collins, 1987; Eaton, 1970; Hayward, O’Brien & Kerley, 2007; Hayward et al., 2007b; Hunter, 1998; Lindsey et al., 2011)	Stein et al., 2012
Site fidelity	Connected protected area patch size < max. observed exploratory movements	(1) Reduce risk of conflict and persecution outside of targeted recipient area (Boast, Good & Klein, 2015; du Preez, 1970; Houser et al., 2011; Marker et al., 2003b; Pettifer et al., 1982; Phiri, 1996)	This study
Urban areas	50 km buffer radius	(1) Safety	Namibia Statistics Agency, 2012

We were not able to include natural prey as a critical element of area suitability (Hayward et al., 2006; Hayward, O’Brien & Kerley, 2007; Lindsey et al., 2011; Winterbach et al., 2015) because standardised wildlife information was not available for most designated PAs. In addition, Namibia’s PA boundaries rarely obstruct wildlife movements effectively and, therefore, local prey densities may fluctuate significantly and according to seasons. Sufficient prey availability needs to be determined case-specifically prior to any future translocations.

Except for Namibia’s coastline, we considered all habitats as potentially suitable because cheetahs used to occur widespread across the country (Gaerdes, 1974; Marker-Kraus et al., 1996). Furthermore, cheetahs inhabit, and readily adapt to, a wide variety of biomes (Bissett & Bernard, 2007; Caro, 1994; Eaton, 1974; Marnewick et al., 2009; Purchase, Vhurumuku & Purchase, 2006; Skinner & Smithers, 1990; Welch et al., 2015), including arid and hyper-arid ecosystems (Mills, 1984; Rostro-García, Kamler & Hunter, 2015; Sunquist & Sunquist, 2002).

We assessed recipient area suitability in a step-wise elimination process—the order of parameter inclusion had no influence on the final results. We computed two different iterations of the model. First, we included all suitability criteria as outlined in Table 2. Secondly, we excluded site fidelity as a critical suitability parameter, reflecting our original study design.

## Results

### Sample characteristics

We investigated the outcomes of 15 cheetah translocations, comprising 23 adults (12 males, 11 females) with 10 dependent offspring, for a total of 7,725 monitoring days (adults only) between May 2008 and October 2014. Translocations involved three cases with livestock depredation backgrounds, 15 indiscriminately captured cheetahs, and five individuals released after long-term captive rehabilitation. Prior to release at an average distance of 419.6 km ± 216.1 km SD (range: 71–816 km), we held cheetahs for 1–1,184 days (350.6 days ± 439.0 days SD, *n* = 23) (Table 1), corresponding with availability of recipient areas, rearing of orphans, presence of offspring, and permit acquisition.

We employed hard releases (*n* = 13) and soft releases (*n* = 10). For soft releases, the median acclimation period at recipient sites was 18.25 weeks (range: 1–38 weeks, *n* = 10). We released cheetahs solitarily (*n* = 5), as mothers with dependent sub-adults (*n* = 4, +10 offspring), as sibling groups (*n* = 7, three events), and as groups artificially bonded during captivity (*n* = 7, three events) (Table 1). We tracked cheetahs with combined GPS satellite—VHF transmitters (*n* = 12), VHF-only transmitters (*n* = 10), while Aju02 had an ID collar as part of a group release. We fitted the GPS unit of Aju40 to Aju41 at a later stage. Based on their habituation to humans (Supplemental Information 2), we classed cheetahs as wild (*n* = 15), semi-habituated (*n* = 4), or fully habituated (*n* = 5) (not tame) at the time of release. All sub-adults released alongside their mothers retained wild characteristics. Except for those individuals released into central Namibia (five adults, three offspring), we released cheetahs (18 adults, seven offspring) into areas where they experienced novel competition with spotted hyaenas.

### Survival

**Figure 2:**
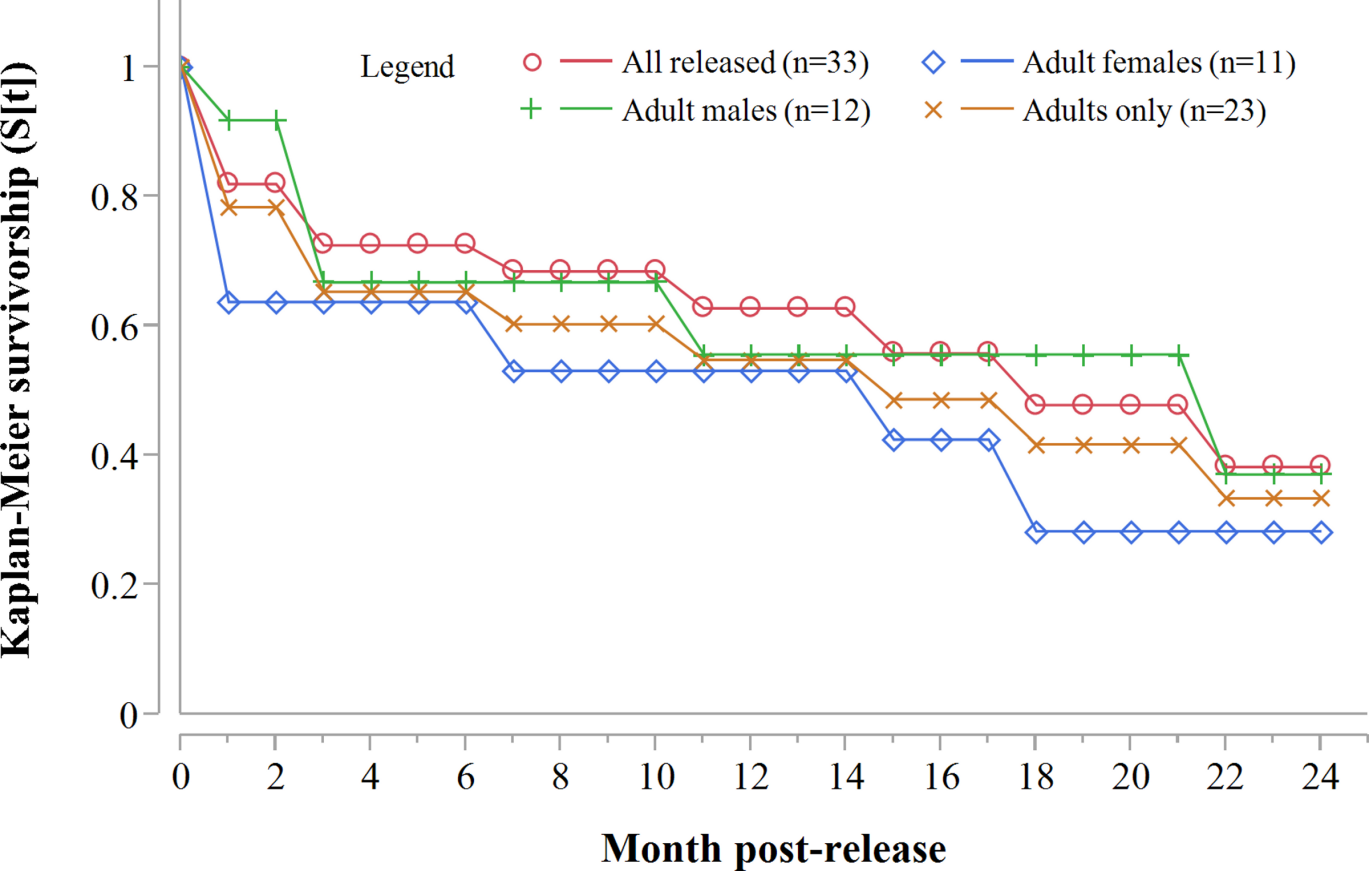
Progressive Kaplan-Meier survivorship estimates for translocated cheetahs in Namibia. Data standardised to month post-release.

Three GPS units failed during the first year. Of the remaining 20 collared adults, 10 died within 12 months of release: eight during the first 71 days. For all collared cheetahs (*n* = 23), the annual Kaplan–Meier survivorship estimate was 0.57 (95% CI [0.35–0.76]) (Fig. 2). Males and females survived equally well (K-M = 0.56, 95% CI [0.26–0.82], *n* = 12; K-M = 0.53, 95% CI [0.23–0.81], *n* = 11 respectively) (*Z* = − 0.1836, *P* = 0.8572).

**Figure 3:**
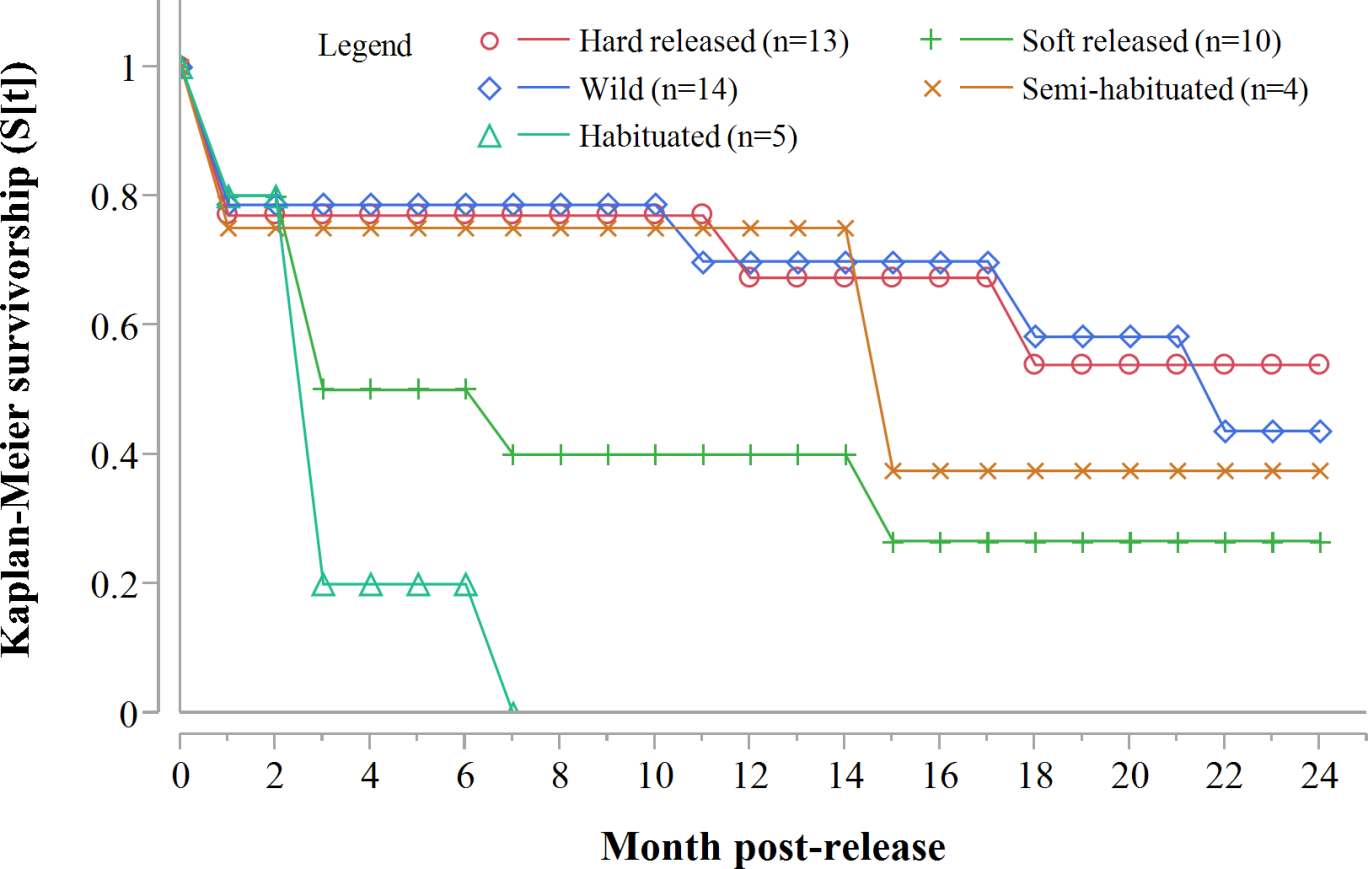
Progressive Kaplan-Meier survivorship estimates for translocated cheetahs by degree of habituation and release mode. Data standardised to month post-release.

Despite acclimation to recipient areas, soft released cheetahs appeared to have lower survivorship in year one (K-M = 0.40, 95% CI [0.14–0.73], *n* = 10) than hard released individuals (K-M = 0.67, 95% CI [0.37–0.88], *n* = 13) (Fig. 3) but not significantly so (*Z* = 1.31, *P* = 0.1902). Fully habituated cheetahs (*n* = 5) had the lowest survivorship (Fig. 3). None of them survived for longer than 205 days. Private landowners (who had not received monitoring data yet) shot four outside recipient areas and a spotted hyaena killed the fifth–giving a median survival period of 2.4 months (range: 0.6–6.8 months, *n* = 5). Landowners who killed habituated cheetahs reportedly shot them because of their lack of fear of humans, rather than for reasons of conflict (Table 3). The association between first year survival and degree of habituation was significant (*χ*^2^ = 8.63, *df* = 2, *P* = 0.0134, *n* = 20).

**Table 3:** Outcomes of cheetah translocations into free-range environments in Namibia.

ID	Known survival (monitoring days)	Homing?	Cause of death	Conflict?	Reproduction	Success?	Comments
Aju01	519	No	Spotted hyaena	No	–	Yes	Stable sibling group
Aju02	426	No	–	No	4 cubs	Yes	Stable sibling group
Aju03	322	No	Natural accident (suspected)	No	Unknown	No	Stable sibling group
Aju07	11	No	Recaptured	No	–	No	Death from acute renal failure (captivity)
Aju17	960	No	–	No	Mating suspected	Yes	–
Aju18	14	No	Shock/Exhaustion	No	–	No	–
Aju19	13	No	Spotted hyaena	No	–	No	Split upon release
Aju20	67	No	–	Unknown	Unknown	Unknown	Split upon release–collar failure
Aju26	112	No	–	Unknown	–	Unknown	Collar failure
Aju29	840	No	–	No	2 cubs	Yes	Split from Aju30 upon release
Aju30	290	No	–	No	–	Unknown	Male accidentally trapped and re-released
Aju34	991	No	Injury–Shot	No	Unknown	Yes	Shot due to severe front leg injury
Aju38	636	No	Spotted hyaena	No	Mating suspected	Yes	–
Aju40	19	No	Spotted hyaena	No	–	No	–
Aju41	205	No	Shot	No	–	No	Killed due to lack of fear of humans
Aju42	71	No	Shot	11 smallstock	–	No	Killed due to lack of fear of humans
Aju43	71	No	Shot	11 smallstock	–	No	Killed due to lack of fear of humans
Aju44	71	No	Shot	11 smallstock	–	No	Killed due to lack of fear of humans
Aju56	680	No	–	No	3 cubs	Yes	–
Aju58	427	No	Shot–livestock carcass	1 cattle calf	5 cubs	Yes	Killed due to livestock depredation
Aju59	19	No	Gin trap	No	–	No	Sub-adults survived after female’s death
Aju65	371	Yes	–	No	Unknown	No	Stable coalition
Aju66	590	Yes	–	No	Unknown	No	Stable coalition

In addition to the early deaths of fully habituated individuals, two spotted hyaenas killed male Aju19 13 days after release. Female Aju07 was re-captured 11 days after release due to apathy. She had become emaciated and dehydrated and succumbed to acute renal failure (I Baines, pers. comm., 2010) >12 months later. After leaving the recipient area, female Aju59 died 19 days post-release due to severe injuries sustained during accidental capture in a gin trap set for control of black-backed jackals (*Canis mesomelas*). Female Aju18 appeared to have died from shock/exhaustion 14 days post-release—a post-mortem delivered no definitive results. She hunted successfully on the day of her release and found at least two permanent water holes on the recipient reserve. Male Aju03 was found with a broken pelvis/spine near a rocky outcrop after almost one year, but the carcass showed no obvious signs of a hyaena attack.

**Figure 4:**
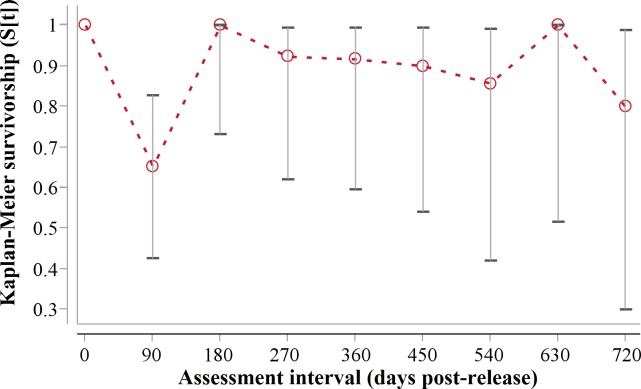
90-day interval Kaplan-Meier survivorship analysis for adult translocated cheetahs. Error bars show 95% CI of the K-M survivorship estimate.

Following three additional mortalities in the second year (Aju01/38/58 in Table 3) and ‘censoring’ of four cases with depleted tracking collars, the progressive Kaplan–Meier survivorship estimate for adults decreased to 0.40 (95% CI [0.21–0.62]) (Fig. 2). Assessed independently across years, adult survivorship in years one (see above) and two (K-M = 0.70, 95% CI [0.35–0.92], *n* = 10) did not differ significantly (*Z* = − 0.703, *P* = 0.4839). However, after the first three months with highest mortality, cheetah survivorship improved and remained ≥0.80 (Fig. 4), suggesting that the initial post-release orientation/exploration period is particularly important in terms of survival. Excluding cases with unknown outcomes, cheetahs surviving the first 90 days (*n* = 12) had an 83% chance of surviving one year, and a 38% chance of surviving for two.

Overall, human persecution (*n* = 7) and spotted hyaena attacks (*n* = 4) had the highest impact on cheetah survival (Table 3). Eight adults died outside of PAs whereas six died on conservation areas. At the end of this study, the mean known survival period of adults was 12.1 months ± 11.1 months SD (range: 0.4–33.0 months, *n* = 20). The survival periods recorded for females (10.5 months ±10.2 months SD, range: 0.4–28.0 months, *n* = 10) and males (13.7 months ± 12.3 months SD, range: 0.4–33.0 months, *n* = 10) did not significantly differ (Wilcoxon-Mann–Whitney *U*-Test: *U* = 58.5, *P* = 0.5443). There was also no significant difference in mean survival periods of hard released cheetahs (14.8 months ± 11.7 months SD, range: 0.4–33.0 months, *n* = 11) and soft released ones (8.7 months ± 10.1 months SD, range: 0.6–28.0 months, *n* = 9) (*U* = 85.5, *P* = 0.5173).

### Reproduction

Of the six females that survived >90 days, four produced litters after settling into permanent ranges. Two females (Aju56/58) successfully reared offspring released with them (*n* = 5) and reproduced again (eight cubs). Females Aju02/29 produced another six cubs (*n* = 6) (Table 3). Median litter size after emergence from lairs was 3.5 (range: 2–5, *n* = 4). Considering a gestation period of ∼94 days for African cheetahs (Brown et al., 1996) and emergence of cubs from the lair at eight weeks of age (Laurenson, 1994), Aju02 conceived as soon as three months post-release. We observed new litters of Aju29/56/58 13–14 months post-release, when cubs appeared to be 2–4 months old (according to phenotypic characteristics described in Eaton, 1969). The known median survival for newborn cubs was 8.0 months (range: 1.0–14.0 months, *n* = 14) and at least 64% reached an age of nine months.

In addition, we observed two males (Aju17/38) during courtship behaviour with wild females, but mating events could not be confirmed.

### Movements

#### Explorations and settling behaviour

**Figure 5:**
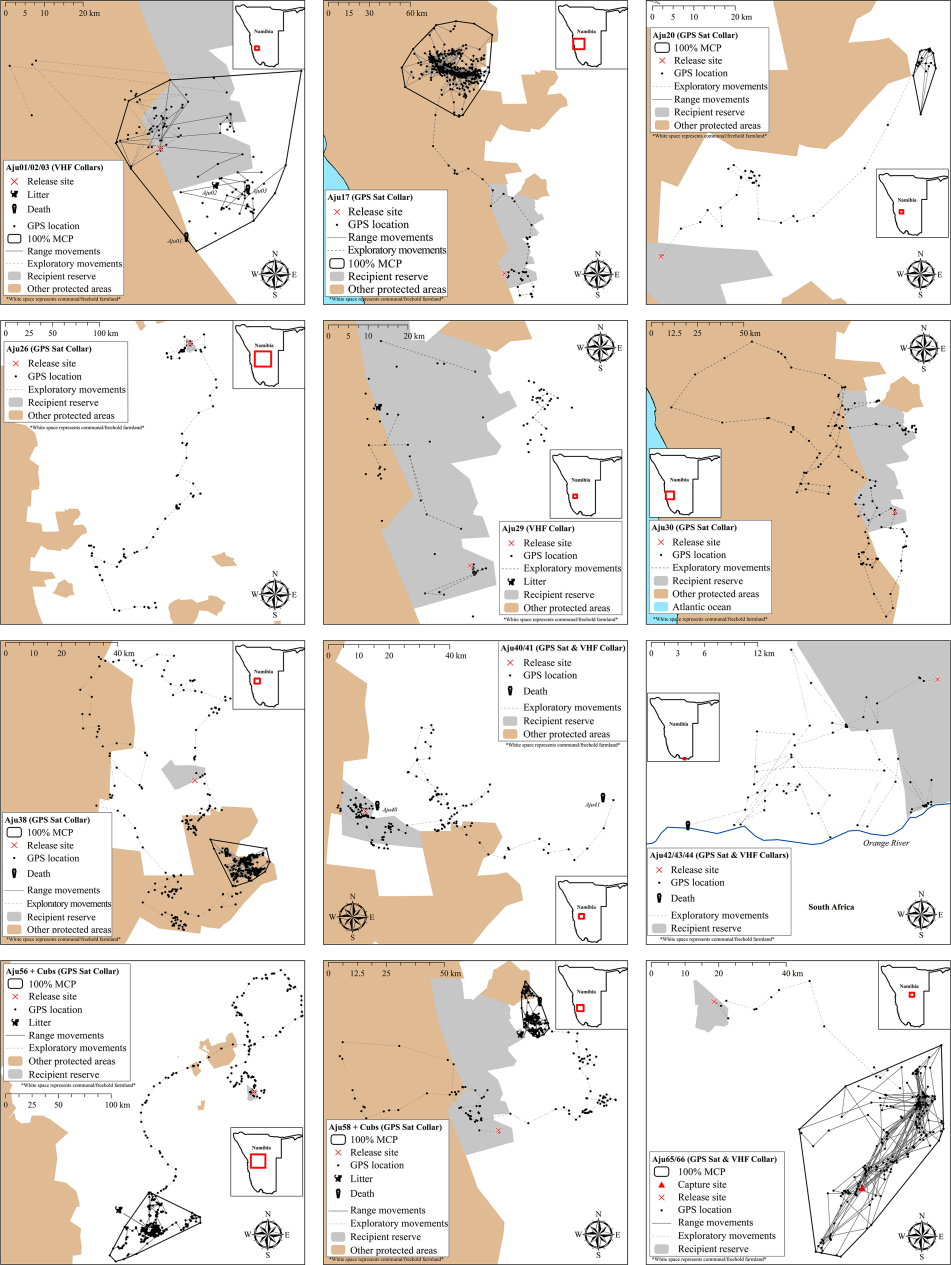
Movements of cheetahs translocated into free-range environments in Namibia. Early deaths and case studies with <60 monitoring locations are excluded. MCP ranges are only displayed for subjects that showed settling behaviour.

**Figure 6:**
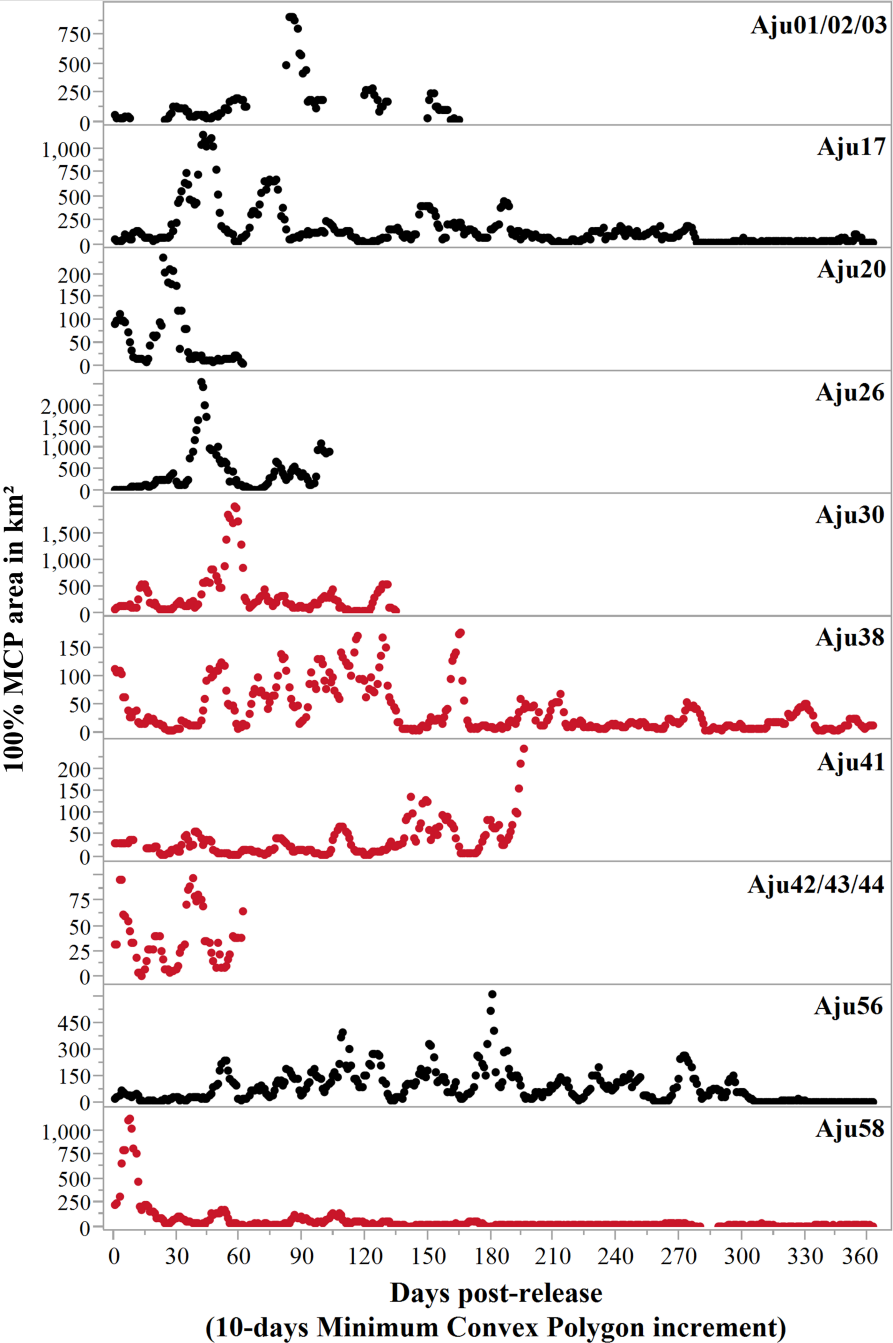
Progressive 100% Minimum Convex Polygon analysis for hard released (black curves) and soft released (red curves) cheetahs. Early deaths (<3 weeks) and case studies with insufficient movement records are excluded. Note: y-axis scales differ between case studies. Area estimates were only calculated if at least five data points were available for 10-day segments.

All translocated cheetahs displayed exploratory movements that extended beyond the boundaries of target recipient reserves and connected PAs (Fig. 5). According to incremental 10-day 100% MCP area progressions, the durations and spatial scales of exploratory movements were highly variable but distinct peaks occurred during the first 6.5 months (Fig. 6). Soft release did not prevent large scale explorations (Fig. 6) which were also not site-specific (Fig. 5).

Measured as the total 100% MCP area utilised, at least five cheetahs roamed >2,000 km^2^ during the first three months after release, and covered >4,000 km^2^ within six months (Table 4). Hard released male Aju26 roamed across 19,743 km^2^ within 112 days (until collar failure) whilst the movements of soft released male Aju30 encompassed 9,048 km^2^ in 180 days. Extensive movements were also not sex-specific. Together with her three sub-adult offspring, Aju56 roamed across 13,596 km^2^ within six months (Table 4). For those cheetahs that established/resumed ranges, settling occurred 13–190 days post-release (median = 93 days) (Table 4), whereas Aju26/30/41/42-44 showed no tendency to settle before collars failed or subjects died (Fig. 6).

**Table 4:** Summary movement statistics for cheetahs translocated into free-range environments.

ID	Linear distance (km) moved in first 3 months (no. of fixes)	100% MCP^a^ area (km^2^) covered		Duration of post- release exploratory movements (days)	Estimated home range size in km^2^			Percent overlap with recipient reserve (all PAs)	Distance (km) of centroid to release site	Distance (km) of last known location to release site
		3 months (no. of fixes)	6 months (no. of fixes)		No. of fixes	100% MCP^a^ (50%)	95% M-KDE^b^ (50%)			
Aju01	Insufficient data	820.2 (54)	1,316.0 (103)	93	92	1,509.0 (284.6)	1,196.3 (108.6)	39 (54)	13.5	24.2
Aju02	Insufficient data	820.2 (54)	1,316.0 (103)	93	92	1,509.0 (284.6)	1,196.3 (108.6)	39 (54)	13.5	17.0
Aju03	Insufficient data	820.2 (54)	1,316.0 (103)	93	92	1,509.0 (284.6)	1,196.3 (108.6)	39 (54)	13.5	24.7
Aju07	n/a	n/a	n/a	n/a	–	n/a	n/a	n/a	n/a	3.9
Aju17	524.1 (89)	7,905.2 (89)	8,373.6 (181)	85	883	3,209.1 (253.9)	931.7 (203.1)	0 (89)	147.1	159.4
Aju18	n/a	n/a	n/a	n/a	–	n/a	n/a	n/a	n/a	38.3
Aju19	n/a	n/a	n/a	n/a	–	n/a	n/a	n/a	n/a	10.9
Aju20	n/a	989.8 (64)	n/a	36	31	49.0 (4.4)	51.8 (6.3)	0 (0)	51.7	81.5
Aju26	590.5 (91)	11,474.1 (91)	n/a	>112	–	n/a	n/a	n/a	n/a	281.0
Aju29	Insufficient data	1,068.5 (46)	1,068.5 (56)	Unknown	–	n/a	n/a	n/a	n/a	9.2
Aju30	577.6 (92)	7,907.3 (92)	9,048.9 (141)	>290	–	n/a	n/a	n/a	n/a	71.0
Aju34	Insufficient data	Insufficient data	Insufficient data	Unknown	–	n/a	n/a	n/a	n/a	24.4
Aju38	265.8 (92)	1,179.9 (92)	3,292.6 (181)	171	418	217.0 (26.9)	121.6 (18.3)	0 (74)	26.1	28.2
Aju40	n/a	n/a	n/a	n/a	–	n/a	n/a	n/a	n/a	4.4
Aju41	147.1 (90)	138.9 (90)	1,099.5 (160)	n/a	–	n/a	n/a	n/a	n/a	78.0
Aju42	n/a	n/a	n/a	n/a	–	n/a	n/a	n/a	n/a	31.9
Aju43	n/a	n/a	n/a	n/a	–	n/a	n/a	n/a	n/a	31.9
Aju44	n/a	n/a	n/a	n/a	–	n/a	n/a	n/a	n/a	31.9
Aju56	250.1 (87)	2,465.0 (87)	13,596.0 (179)	190	497	2,624.8 (94.6)	929.5 (81.5)	0 (0)	118.1	140.6
Aju58	363.0 (91)	2,520.1 (91)	4,694.1 (181)	114	318	176.8 (18.6)	138.7 (13.9)	0 (14)	41.7	59.2
Aju59	n/a	n/a	n/a	n/a	–	n/a	n/a	n/a	n/a	26.0
Aju65	582.6 (91)	1,877 (91)	2,015.0 (165)	13	275	1,614.4 (370.1)	1,182.2 (193.2)	0 (0)	64.7	71.1
Aju66	582.6 (91)	1,877 (91)	2,015.0 (165)	13	275	1,614.4 (370.1)	1,182.2 (193.2)	0 (0)	64.7	65.2

**Notes.**

a MCP, Minimum Convex Polygon

b M-KDE, Movement-based Kernel Density Estimator

Neither mountain escarpments with 500–600 m elevations (often at near vertical inclines) nor true desert environments limited exploratory movements of cheetahs. Seven adults traversed large areas of the hyper-arid Namib Desert (see Aju30 in Fig. 5). Male Aju17 established a permanent range encompassing sand dunes whilst female Aju29 eventually raised cubs in this environment.

### Homing

**Figure 7:**
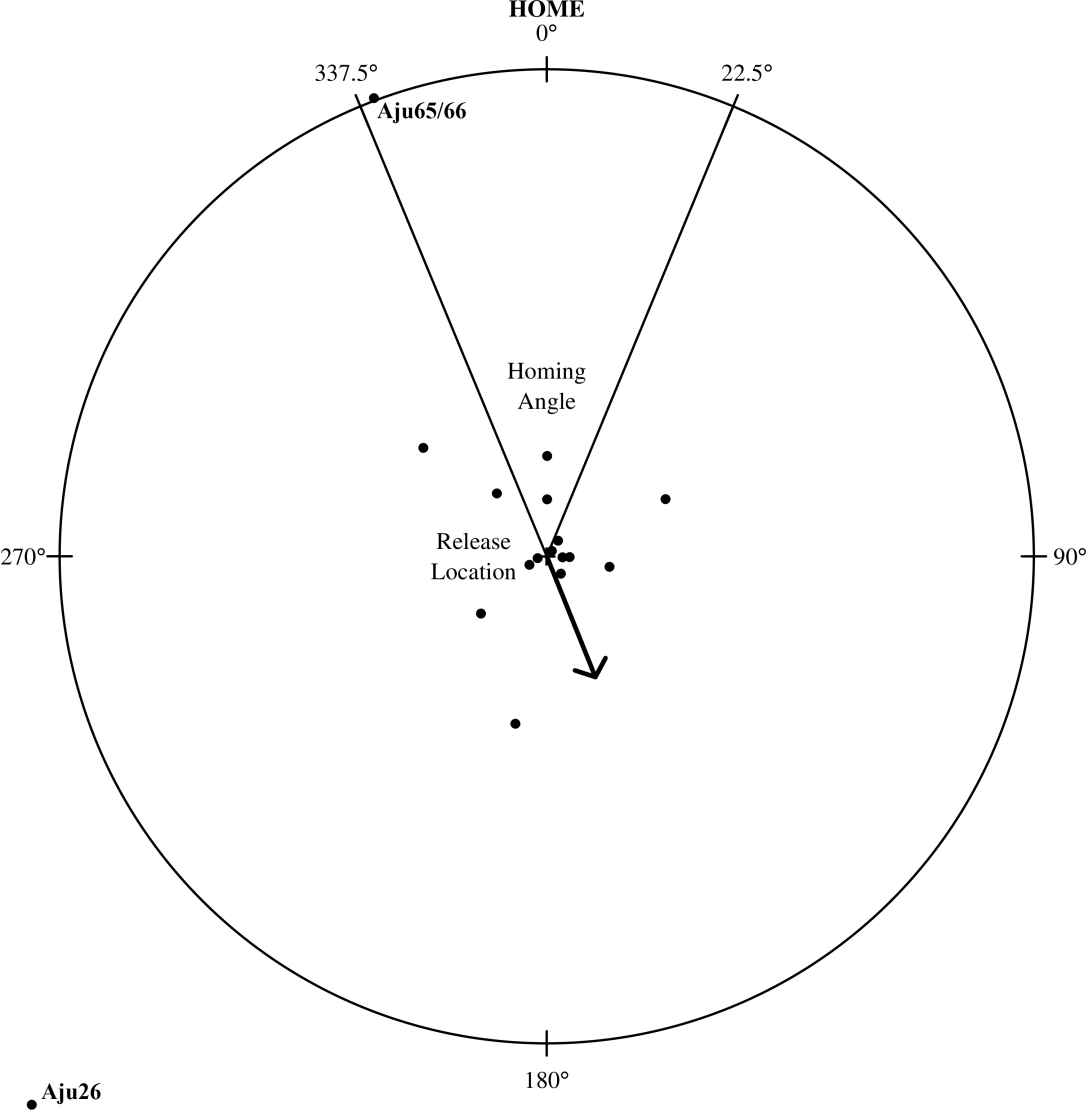
Homing assessment for 23 translocated cheetahs relative to their capture location. The distance from the circle’s centre (individual’s release location) to outer edge represents an individual’s distance travelled relative to its capture location. The arrow shows mean angle (158.1° ± 27.8°SE) for all individuals and stable groups (*n* = 17).

Despite extensive explorations, only the shortest translocation (Aju65/66 translocated 71 km) resulted in successful homing (Fig. 7). The male coalition returned to the capture property and was assumed to resume its previous home range within two weeks (Fig. 5). All cheetahs translocated >137 km did not home and there was no evidence of oriented, directional movements towards capture sites (Fig. 7). Cheetahs also did not return to the captive facility. The mean distance of last monitoring locations to release sites (Table 4) was 57.1 km ± 63.2 km SD (*n* = 23 adults) and therefore >14% of the average translocation distance. For non-homing cheetahs (*n* = 21), the mean distance of last locations to capture sites was 454.1 km ± 197.8 km SD (range: 153.2–826.7 km).

The mean direction of an individual’s last location in relation to release site deviated 173.5° ± 106.2°SD (*n* = 23) from true home. When analysed in 90°segments around the release centroid, the distribution of directions in relation to true home was random (*χ*^2^ = 0.13, *df* = 3, *P* = 0.988, *n* = 23), suggesting that cheetahs did not orient toward any particular geographic direction. The distribution of angles located within and outside of the homing sector (i.e., four = in, 19 = out) also significantly differed from the distribution that would be expected if all cheetahs had homed (*χ*^2^ < 0.01, *df* = 1, *P* < 0.0001, *n* = 23). Neither translocation distance (*R*^2^ = 0.059, *P* = 0.269, *n* = 23) nor time in captivity (*R*^2^ = 0.012, *P* = 0.6124, *n* = 23) were strongly associated with an individual’s post-release orientation.

### Range areas

We monitored 14 cheetahs for >90 days and 10 settled into range patterns (Fig. 5), including homing males Aju65/66. Hence, only 35% of released individuals established new ranges. Although Aju29/34 appeared to have settled successfully (Fig. 5), telemetry data were too sporadic to confirm this. The GPS units of Aju26/30 failed before these individuals showed settling behaviour.

Range sizes (excluding Aju65/66) in semi-arid to hyper-arid areas of south-western Namibia varied between 49.0–3,209.1 km^2^ (Table 4). The average distance of geometric range centres (the mean x,y coordinates of range data) to release sites was 66.4 km ± 53.7 km SD (range: 13.5–147.1 km, *n* = 6). Only the range of Aju01–03 overlapped with the recipient reserve whereas two ranges did not overlap with any PA and four had partial overlap with other conservation areas (Fig. 5). Average percent overlap with all PAs was 38.5% ± 39.0% SD (range: 0–89%) and ranges appeared stable until monitoring ceased.

### Site fidelity

Due to extensive explorations, site fidelity was low (Table 5). Mean overlap of daily locations with recipient reserves in year one was 39.8% ± 7.2% SE (*n* = 23). All cheetahs left recipient reserves and moved into free-hold farmland areas and/or into other private and public conservation areas (Fig. 5). Site fidelity increased significantly when defined to include occupancy of other PAs (Table 5), demonstrating the importance of PA connectivity to improve the safety of far-ranging carnivores released into unfenced environments.

**Table 5:** Percent site fidelity of translocated cheetahs. Results represent data for 12 months post-release.

Category	No. of locations (*n*)	A—All conservation area				B—Recipient reserves only				Comparison A vs. B
		Min	Mean (SE)	Max	Total	Min	Mean (SE)	Max	Total	Wilcoxon signed rank test
All adults (*n* = 23)	3,199	1.04	54.89 (7.49)	100.00	44.45	0.82	39.80 (7.16)	100.00	16.91	*S* = 27.5, *P* = 0.001^*^
Males (*n* = 12)	1,916	1.04	50.29 (10.61)	100.00	50.26	0.82	29.25 (8.47)	100.00	12.21	*S* = 5.0, *P* = 0.0625
Females (*n* = 11)	1,283	4.69	59.90 (10.87)	100.00	35.78	3.58	51.30 (11.15)	100.00	23.93	*S* = 10.5, *P* = 0.0156^*^
Soft released (*n* = 10)	1,340	26.65	54.26 (8.18)	100.00	53.34	0.82	36.95 (8.75)	100.00	19.33	*S* = 7.5, *P* = 0.0313^*^
Hard released (*n* = 13)	1,859	1.04	55.36 (11.98)	100.00	37.98	1.04	41.99 (11.01)	100.00	15.17	*S* = 7.5, *P* = 0.0313^*^
GPS collars (*n* = 12)	2,336	1.04	46.18 (10.71)	100.00	44.65	0.82	24.78 (8.65)	100.00	10.83	*S* = 10.5, *P* = 0.0156^*^
VHF collars (*n* = 11)^a^	863	1.04	64.38 (10.16)	100.00	47.76	1.04	56.18 (9.73)	100.00	33.37	*S* = 5.0, *P* = 0.0625

**Notes.**

a Including female Aju02 with ID collar.

* Denotes statistical significance at *α* = 0.05.

Site fidelity of males and females (Table 5) did not differ significantly when assessed for recipient reserves only (*U* = 9.5, *P* = 0.1875) and for all PAs (*U* = 2.5, *P* = 0.4102). Likewise, site fidelity did not differ between hard and soft released animals (for recipient reserves and all PAs: *U* = − 9.5, *P* = 0.8555). There was also no difference in the mean number of days that hard released (19.0 days ± 15.4 days SD, *n* = 12) and soft released (19.7 days ± 23.1 days SD, *n* = 10) cheetahs spent within a 10 km radius around release sites (*U* = 79.0, *P* = 0.7069). For soft released individuals, time spent in acclimation pens did not significantly influence site fidelity on recipient reserves (Spearman’s *R_s_* = 0.331, *P* = 0.3498, *n* = 10).

Cheetah site fidelity results were also not significantly influenced by recipient area size (*R*^2^ = 0.0738, *P* = 0.2099, *n* = 23). Mean percent overlap of positional data with recipient areas was higher for cheetahs released onto reserves measuring >700 km^2^ (45.7% ± 29.5% SD, *n* = 13) than for those released onto reserves <350 km^2^ (32.1% ± 40.1% SD, *n* = 10) but did not differ significantly (*U* = 117.0, *P* = 0.0992). This suggests that even the largest private PAs (e.g. NamibRand in Fig. 1) cannot contain extensive post-release movements reliably. Of all cheetahs monitored >90 days (*n* = 14), eight left recipient reserves permanently whilst six returned sporadically. Neither time in captivity (*R*^2^ = 0.012, *P* = 0.6128, *n* = 23) nor translocation distance (*R*^2^ = 0.012, *P* = 0.6129, *n* = 23) significantly influenced site fidelity results.

Although deployed at near-equal rates (GPS units = 12, VHF/ID units = 11), VHF units provided <27% of positional data in year one. However, the site fidelity estimate for VHF-tagged subjects (recipient reserves only) was more than twice as high as that for GPS-tracked cheetahs (Table 5). We could not detect VHF-tagged cheetahs for prolonged periods (range: 1–253 days), resulting in 124 data gaps in year one (at least one day missing between subsequent locations) with a mean gap interval of 11.0 days ± 34.8 days SD. Conversely, GPS collars produced only 37 data gaps and the mean gap interval (3.4 days ± 4.6 days SD, range: 1–22 days) was significantly shorter (*T* = 2.36, *P* = 0.0098). Considering this impact of tracking technology on detection probability, true cheetah site fidelity probably was <25% on recipient reserves, and <47% on all PAs (category GPS in Table 5).

### Prey and conflict

**Figure 8:**
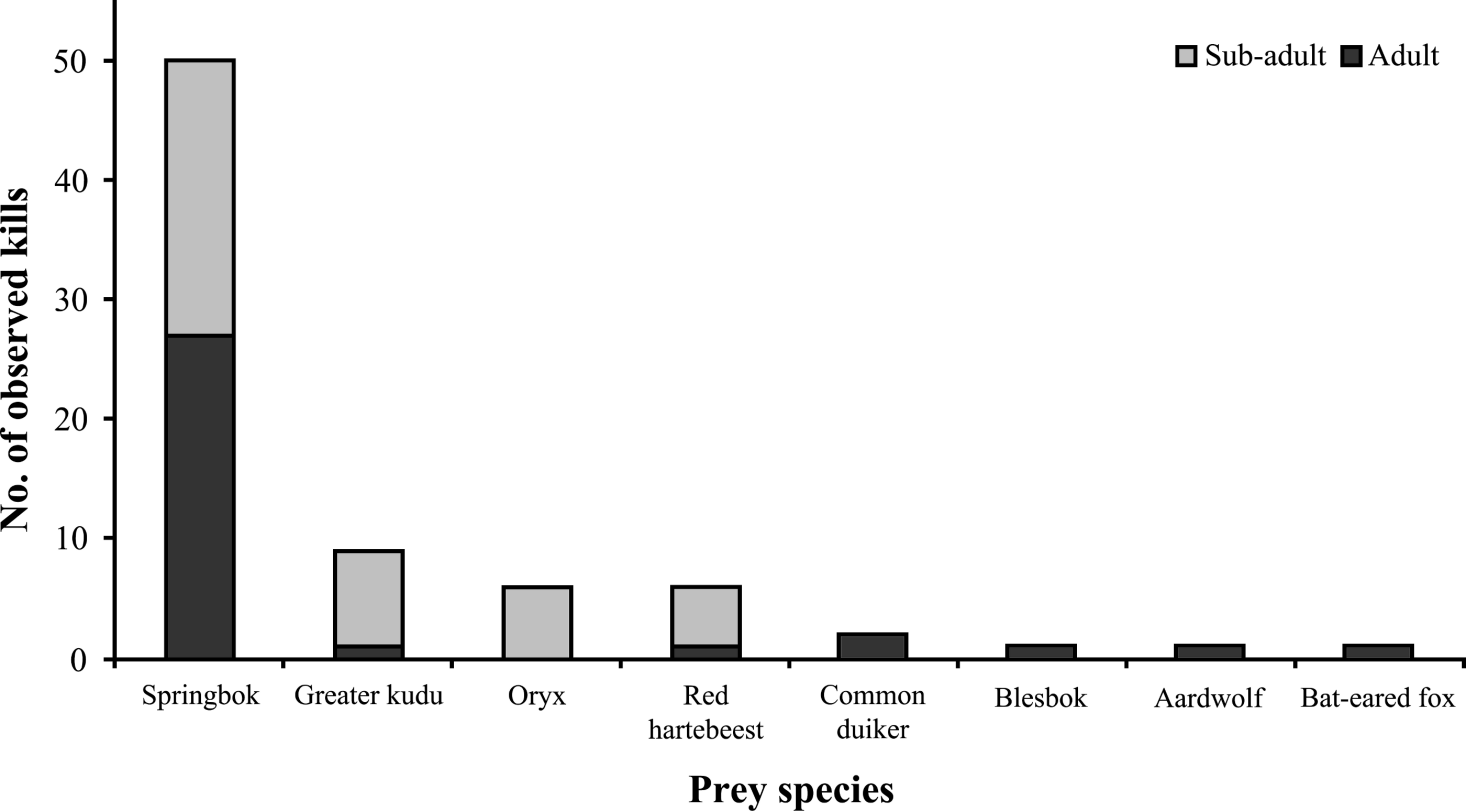
Documented wildlife prey of translocated cheetahs. Kills (*n* = 76) represent direct observations and carcasses located through VHF/GPS telemetry.

Medium to large ungulates comprised the bulk of known wildlife prey (*n* = 76) (Fig. 8), with springbok (*Antidorcas marsupialis*) being the preferred prey species (66% of kills). Cheetahs killed approximately similar proportions of sub-adult and adult ungulates (*χ*^2^ = 1.351, *df* = 1, *P* = 0.245, *n* = 74) and showed no preference for different springbok age classes (*χ*^2^ = 0.32, *df* = 1, *P* = 0.572, *n* = 50). Female Aju18 hunted as soon as ∼8 h after release, whereas Aju07 did not begin to hunt for 11 days, resulting in her re-capture.

Except for two case studies involved in confirmed livestock predation (the damage was compensated), data sharing with land managers showed that translocated cheetahs caused little conflict (Table 3). During explorations, however, cheetahs frequently roamed across multiple farmland properties within a 24 h period, making effective data sharing impossible. It remains unknown whether they killed livestock during these periods. Farmers from whose properties known livestock raiders had been moved (*n* = 2) reported new conflict with cheetahs 12–24 months later, showing that translocation was not a permanent solution. Due to continued perceived threats to valuable game (*n* = 5) and livestock (*n* = 4), the majority of managers (64%) from whose properties cheetahs were translocated (*n* = 14) requested repeat removals within two years of first events, giving rise to concerns that sustained translocations could locally induce source sink effects.

### Success

**Figure 9:**
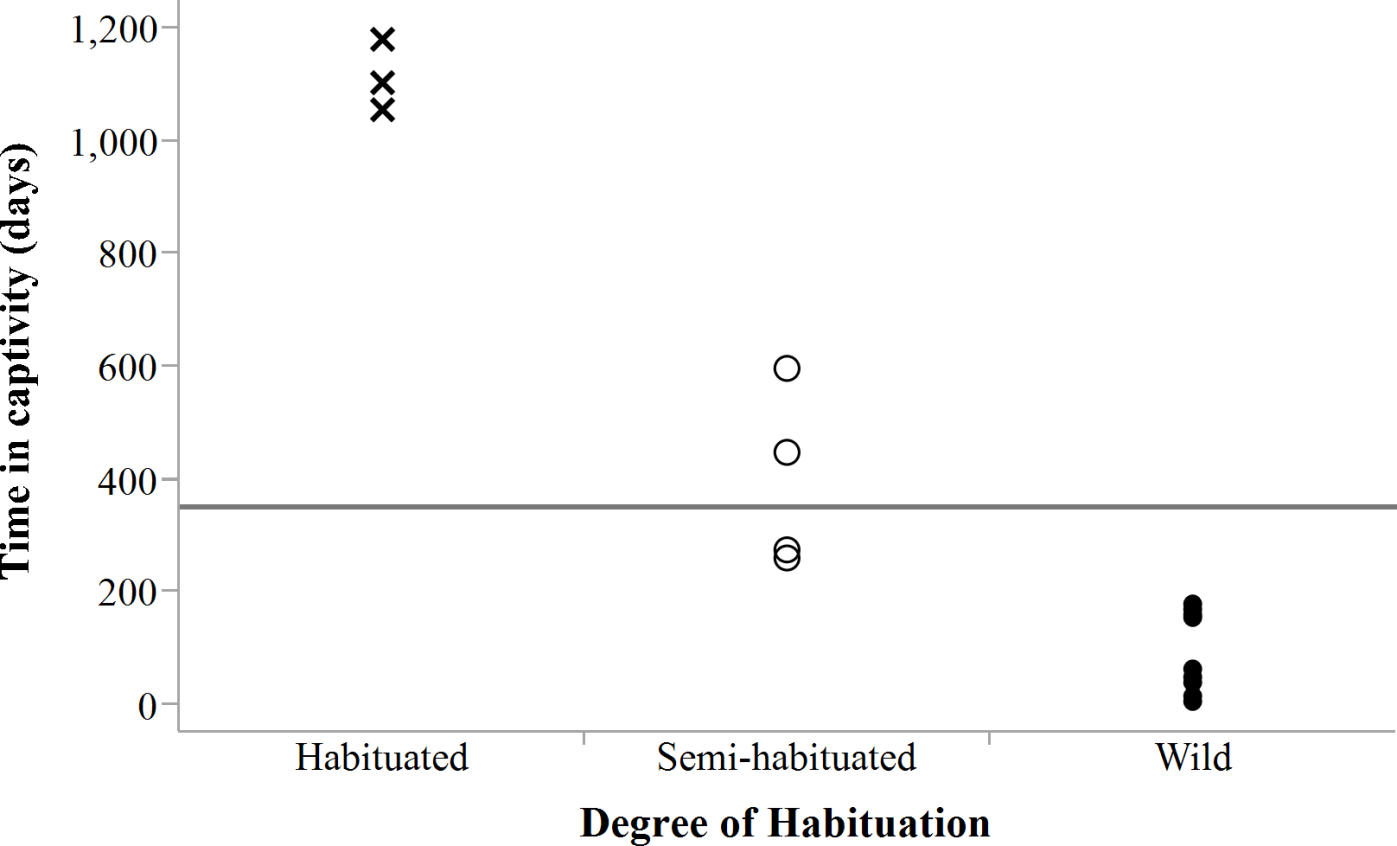
Degree of habituation to humans exhibited by cheetahs in relation to time in captivity. Horizontal line shows mean captive time (350.6 days ± 439.0 days SD) for all individuals (*n* = 23). Note: a datum may represent multiple individuals held captive for the same period.

Success evaluations were possible for 20 translocated adults (Table 3), giving an overall success rate of 40%. Translocation success was not significantly associated with sex (*χ*^2^ = 0.84, *df* = 1, *P* = 0.3593, *n* = 20) or release mode (*χ*^2^ = 1.29, *df* = 1, *P* = 0.2526, *n* = 20) but with degree of habituation to humans (*χ*^2^ = 6.47, *df* = 2, *P* = 0.0394, *n* = 20). The latter was strongly associated with the amount of time that cheetahs spent in captivity (LogLikelihood = 21.58, *χ*^2^ = 43.154, df = 2, *P* < 0.0001, *n* = 23), suggesting that animals intended for free-range release should not be raised in captivity and ideally be held <250 days (Fig. 9) to increase their chances of survival in anthropogenic landscapes. Spotted hyaena related mortality (*n* = 4), as another important factor influencing survival, accounted for 33% of confirmed deaths (*n* = 12) of adults translocated into areas with new competition (*n* = 18).

All translocations into the far South of Namibia were unsuccessful and were stopped after four early deaths. Conversely, cheetahs in the southwestern pro-Namib ecosystem (*n* = 12 with known outcomes) and central Namibia (*n* = 4 with known outcomes) were equally successful (50% respectively). Translocations were carried out at a median cost of $2,760 per cheetah (range: $269–$7,559, *n* = 23), giving an Individual Conservation Cost of $6,898 per successful adult (see details in Weise, Stratford & van Vuuren, 2014).

### Potential cheetah recipient areas in Namibia

Including all parameters to determine recipient area suitability in Namibia (Table 2), our model found no suitable PAs for cheetah releases. Due to extensive exploratory movements (Fig. 5; Table 4), site fidelity had the highest individual parameter impact on area suitability (Table 6), eliminating 95% of available public and private PAs. Currently few unfenced PAs exist that are large enough to prevent cheetahs from re-entering commercial or communal farmlands.

**Table 6:** Parameter impact on cheetah recipient area suitability in Namibia.

Exclusion criterion (Table 2)	Suitable area (km^2^) after exclusion from available PAs (302,580.9 km^2^)	Percent of available PAs eliminated	Display
High and zero cheetah occurrence	81,157.0	73.2	Supplemental Information 5
Medium–high spotted hyaena occurrence	86,883.0	71.3	Supplemental Information 6
Medium–high lion occurrence	176,739.5	41.6	Supplemental Information 7
Urban safety buffer	269,902.8	10.2	Supplemental Information 8
Site fidelity (all translocated cheetahs)	15,071.3	95.0	Fig. 10

**Figure 10:**
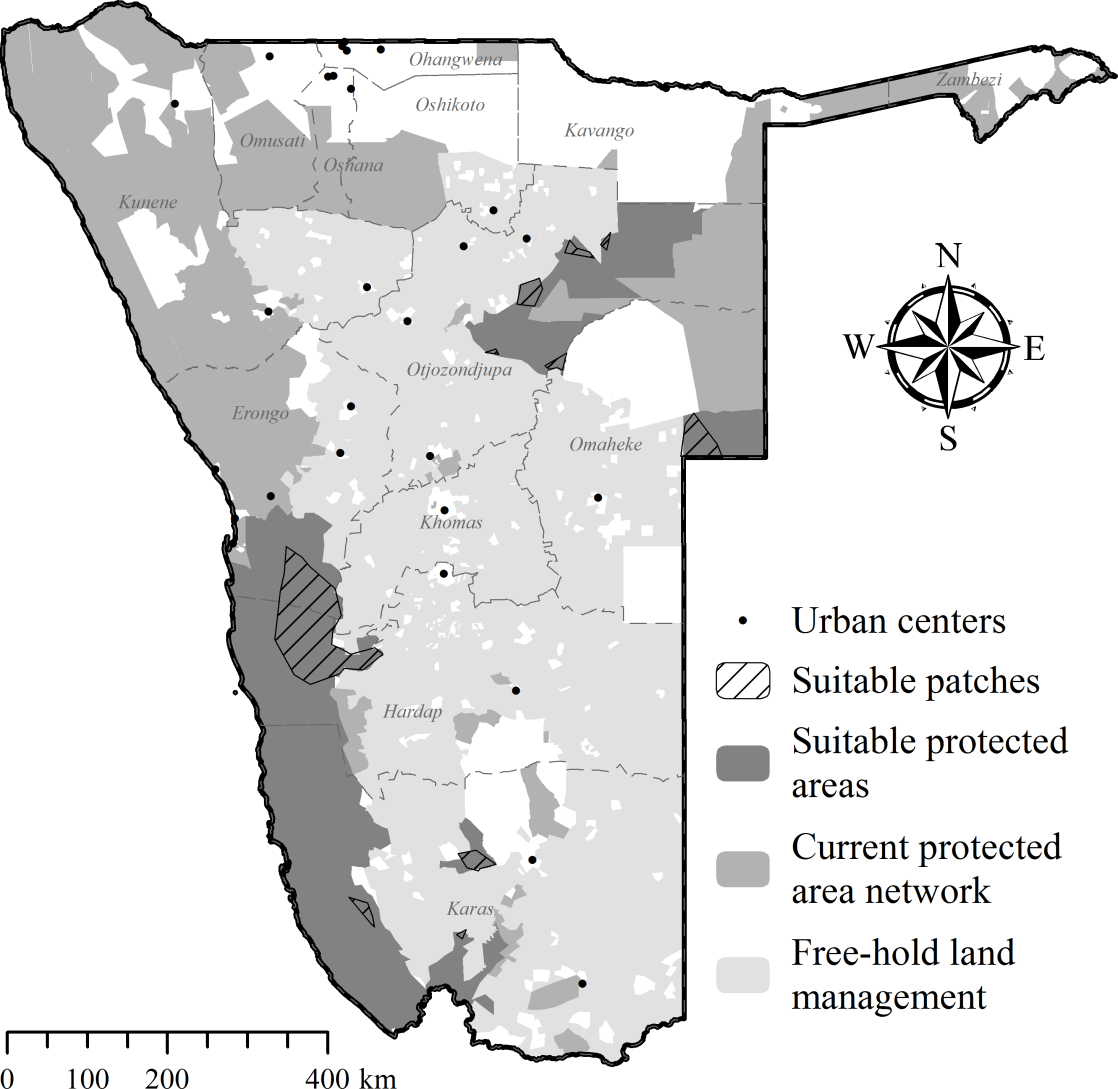
Potential recipient areas for cheetah translocations in Namibia without site fidelity considerations. Further details of areas identified as potentially suitable are provided in Supplemental Information 9.

Excluding site fidelity as a prerequisite, CaTSuiT identified 10 isolated patches (Fig. 10) that fulfilled suitability criteria (individual modelling steps in Supplemental Information 4–Supplemental Information 8). Of these, nine patches are <1,800 km^2^ and six patches are <400 km^2^ (Supplemental Information 9), implying that most cheetahs would leave suitable patches within three months of release (see Table 4) and thus be exposed to pre-translocation threats. Even the largest available patch (10,785.3 km^2^ in western Namibia— Fig. 10) was smaller than the observed post-release movements of at least two case studies (Fig. 5; Table 4). In addition, several translocated cheetahs have already moved into the largest predicted patch, and following successful reproduction on nearby reserves (females Aju02/29/58– Table 3), translocations into the pro-Namib ecosystem were stopped in 2012 because an increasing frequency of cheetah observations suggests recovery of the local population (Q Hartung, N Odendaal, S Bachran, pers. comm., 2014).

## Discussion

The outcomes of cheetah translocations were strongly polarised. Cheetahs either died soon after release, or they survived and integrated into novel environments with good chances of contributing to the free-ranging gene pool. Common problems associated with large carnivore translocations, such as homing and post-release conflict (Linnell et al., 1997), did not significantly influence success. Long-distance translocations proved effective in preventing homing, but survival rates mirrored those of farmland cheetahs in northern-central Namibia (Marker et al., 2003a), suggesting similar pre- and post-translocation mortality. In support of this, Marker et al. (2003a) found no significant difference in survivorship of cheetahs released at the capture site, those translocated less than 100 km, and those moved 100–600 km.

Intra-guild competition with spotted hyaenas had a marked effect on survivorship (also see Hayward et al., 2007a; Marnewick et al., 2009) and lion and spotted hyaena densities are likely to be key predictors of cheetah translocation success (Purchase, Vhurumuku & Purchase, 2006). In this study, most cheetahs came from areas without spotted hyaena populations and hence probably were naïve about the dangers of competition (Jule, Leaver & Lea, 2008; Marnewick et al., 2009). Human-related mortalities, however, had the highest impact on cheetah survival after animals left target recipient areas. Fully habituated cheetahs were particularly unsuccessful, rendering re-wildling of captive-reared cheetahs an unsuccessful strategy (also see Hunter & Rabinowitz, 2009; Pettifer, 1981), and one that incurs the highest costs (Houser et al., 2011; Weise, Stratford & van Vuuren, 2014). Translocating cheetahs into managed reserves that provide control over movements and large carnivore assemblies can mitigate the influence of humans and competing predators, thus improving prospects of survival considerably (Marnewick et al., 2009).

Contrary to others (e.g. Hunter, 1998; Pettifer et al., 1982), we did not observe a beneficial effect of soft release to reduce exploratory movements, even after acclimation of up to 9.5 months. There are many potential reasons for large-scale explorations. For example, they may reflect an individual’s attempt to home or to familiarise with resources in novel environments. For southern African cheetahs, mixed habitat structures were linked with reduced kleptoparasitism, flexible hunting strategies, and security (e.g. Bissett & Bernard, 2007; Broomhall, Mills & du Toit, 2003; Mills, Broomhall & du Toit, 2004; Rostro-García, Kamler & Hunter, 2015; Welch et al., 2015), thus providing suitable ecological niches (Durant, 1998a; Purchase, Vhurumuku & Purchase, 2006). Vegetation cover is particularly important for females to increase cub survival (Durant, 1998a; Durant, 1998b). Here, translocated cheetahs originated from areas with medium to high woody vegetation cover but we released most individuals into sparsely vegetated desert environments, possibly exacerbating movements in search of familiar habitat conditions. Erratic post-release movements could also indicate reactive avoidance behaviour following encounters with spotted hyaenas (Broekhuis et al., 2013) and the cheetah’s strong dependence on migratory prey (here springbok) can influence range areas (Broomhall, 2001; Durant et al., 1988). In some cases, explorations may simply reflect the ecological status of individuals. Long-term research has demonstrated that most cheetahs are not strictly territorial (Caro, 1994; Eaton, 1974; J Melzheimer, pers. comm., 2015). Therefore, certain individuals (like male floaters Caro, 1994) may not show post-release settling behaviour. Under free-range conditions, the magnitudes and complexity of these factors would be very difficult to control, resulting in a high degree of uncertainty with regard to cheetah translocation outcomes.

Additional complications arise from concerns over fitness-related disadvantages of translocated individuals due to local variations in pathogen exposure and associated immune responses and adaptations in Namibian cheetahs (e.g. Castro-Prieto et al., 2012; Thalwitzer et al., 2010). Moreover, translocation of males into extant range areas could cause increased mortality and competitive exclusion through intra-sexual aggression with established residents (Caro & Collins, 1987; Eaton, 1970; Eaton, 1974; J Melzheimer, pers. comm., 2015).

Despite apparent failures in translocating cheetahs, several females successfully raised cubs released with them and surviving females produced new litters. These successes partially compensated for initial losses, and a resident cheetah population established in the pro-Namib desert transition zone (Q Hartung, N Odendaal, pers. comm., 2014) where we released most individuals. From the limited data available, it appears that translocated cheetahs mainly hunted medium-sized prey, including sub-adults of large ungulates. Therefore, detected prey items were in strong agreement with the food selection of Namibian conspecifics (e.g. Marker et al., 2003c; Morsbach, 1986) and prey preferences of the species in general (Hayward, 2009; Hayward et al., 2006). The fact that only few individuals were involved in livestock predation shows that cheetahs do not preferentially select for domestic animals where wild ungulate prey is available (Marker et al., 2003c; Morsbach, 1986; Voigt et al., 2014) and that conflict is not a default outcome of carnivore translocations (Weise et al., 2015). Cheetahs also supported local tourism efforts on recipient reserves and on farms where they settled. Managers received regular position updates that enabled direct observations by guests, thereby attaching tangible value to cheetahs that are highly sought-after tourism attractions (Lindsey et al., 2007; Maciejewski & Kerley, 2014).

**Figure 11:**
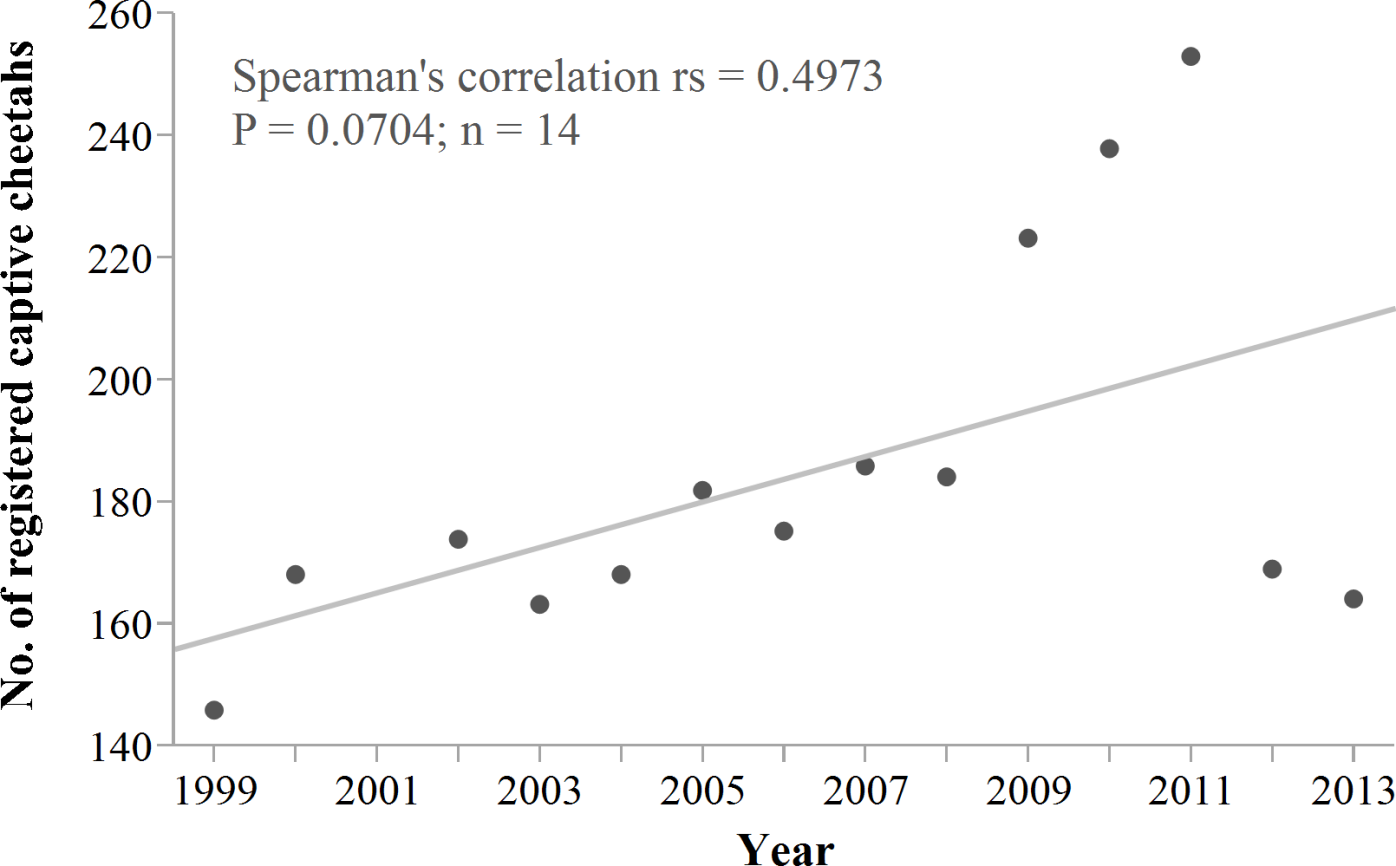
Namibia’s registered captive cheetah population 1999–2013. Trend line shows robust fit. The sudden drop in 2012/2013 probably is an effect of reduced reporting rather than an actual decrease in captive numbers. Data source: International Cheetah Studbook (1999–2013); Cheetah Conservation Fund, Otjiwarongo, Namibia.

Cheetah translocations probably are most cost- and conservation-effective where they can contribute to structured reintroductions of the species (Hayward et al., 2007a; Hayward et al., 2007b; Marnewick et al., 2009; Purchase, Vhurumuku & Purchase, 2006) and do not compromise the viability of the source population. However, reintroductions require large fenced areas and local prey supplementation (Lindsey et al., 2011). Successful establishment of isolated populations on fenced reserves also necessitates artificial meta-population management (Lindsey et al., 2009; Marnewick et al., 2009). There are currently few fenced reserves in Namibia (Jones, 2014) that could receive cheetahs, and if so, only at limited carrying capacities (Hayward, O’Brien & Kerley, 2007; Hayward et al., 2007b). Namibia’s wildlife authorities also stopped the live export of conflict cheetahs, a policy used to supplement reintroduction efforts elsewhere in southern Africa in the past (Hunter, 1998; Phiri, 1996; Marker et al., 2003b). Furthermore, live removals from conflict farms have already resulted in a substantial increase in Namibia’s captive cheetah population (Fig. 11). Some carnivore programmes stopped rescuing cheetahs due to the limited capacity of fenced reserves to accommodate a persistent influx (T Hoth, pers. comm., 2014).

So, where do we go with conflict cheetahs in the future? At first glance, Namibia appears to provide favourable conditions to manage trapped cheetahs by means of free-range translocations. These include a formal PA network covering >35% of the country, suitable prey populations within and outside of PAs (e.g. East, 1999; Lindsey et al., 2013b) and a low human population density of <1.0 people per km^2^ in most areas (Mendelsohn, 2006). However, our modelling of recipient areas shows that the capacity for continued translocations into unconfined environments is very limited and probably far less than the potential demand. The observed post-release dispersals rendered most PAs too small to ensure relative safety of cheetahs. Cheetahs translocated under similar circumstances in Botswana had even lower success (∼18%); the authors mainly attributed failures to low site fidelity and homing that exposed cheetahs to persecution (Boast, Good & Klein, 2015). Extensive movements away from target recipient PAs are a universal issue concerning large carnivore translocations and any of the species involved (Linnell et al., 1997; Massei et al., 2010). Consequently, the challenges of identifying suitable recipient areas and successful protocols apply not only to the cheetah, or to any specific country. If unfenced release sites cannot contain explorations, promoting coexistence of predators and people in unprotected multi-use landscapes will be more effective than relocating animals.

Our results corroborate the fact that translocations rarely resolve perceived conflict on source properties permanently (Boast, Good & Klein, 2015), resulting in repeat requests for live removals. Hence, continued translocations may support removal of cheetahs from their indigenous areas, contradicting the general objective of maintaining a viable free-ranging population in Namibia (Nowell, 1996). In South Africa, the potential population sink effect of sustained translocations from conflict farms into protected reserves was one of the main reasons to cease a successful relocation programme (K Marnewick, pers. comm., 2015). Namibia supports the largest number of cheetahs in any country (Marker, 2005; Purchase et al., 2007). Given the diverse constraints identified in this study, translocations can successfully conserve individual cheetahs and help boost low-density populations where land-use has changed and supports the species’ presence. The actual conservation task, however, remains to ensure the perseverance of an entire population on free-hold farms. With a view on conflict mitigation, alternative options are available (e.g. Ogada et al., 2003; Potgieter, Kerley & Marker, 2015; Schumann et al., 2006). These can provide proactive help rather than a symptomatic treatment. Research in South Africa demonstrated that livestock protection can be cheaper than continued carnivore persecution (McManus et al., 2014), providing an economic incentive for conflict-affected farmers to adopt preventative action in the future. Based on our work with over 350 Namibian land managers since 2008, we emphasise that there is great potential for rapid conflict response coupled with participatory monitoring of resident cheetahs to increase local tolerance of the species and actively engage managers in their *in situ* conservation (also see Bangs et al., 2006; Wachter et al., 2006).

## Conclusions

Translocation can successfully conserve individual cheetahs and locally boost their populations. However, Namibia currently does not provide sufficient environments for the relocation of large numbers of trapped cheetahs. Hence, our findings confirm those from other areas where translocation was tested to manage perceived conflict cheetahs (e.g., Boast, Good & Klein, 2015; Marnewick et al., 2009; Purchase, Vhurumuku & Purchase, 2006). Although with varying levels of local success, the cumulative results from cheetah translocation studies strongly suggest that the strategy should be reserved as a last-resort, supplementary management tool when other conservation options have been exhausted and predominantly for those populations facing imminent local extinction, such as the critically endangered Asiatic cheetah (*A. j. venaticus*) (Durant et al., 2008). Where translocation is necessary, rigorous candidate and recipient area selection can improve its efficacy, thereby justifying the high logistic demands and financial costs. Finally, there is increasing evidence that translocation does not curb the motivation of land managers to remove cheetahs repeatedly, thus fuelling a psychology that is counter-productive to improving coexistence and the maintenance of free-ranging cheetah gene pools.

## Supplemental Information

10.7717/peerj.1346/supp-1 Supplemental Information 1 Additional detail for captivity, immobilisation, transport, and data sharing protocols for translocated cheetahs in Namibia

10.7717/peerj.1346/supp-2 Supplemental Information 2 Classification used to determine degree of habituation of translocated cheetahs

10.7717/peerj.1346/supp-3 Supplemental Information 3 Key characteristics of cheetah recipient reserves

10.7717/peerj.1346/supp-4 Supplemental Information 4 Protected area network (302,580.9 km^2^) potentially available for cheetah translocations in Namibia–CaTSuiT input area

10.7717/peerj.1346/supp-5 Supplemental Information 5 Cheetah occurrence in Namibia (according to Stein et al., 2012) and suitable protected area patches with low–medium occurrence

10.7717/peerj.1346/supp-6 Supplemental Information 6 Spotted hyaena occurrence in Namibia (according to Stein et al., 2012) and suitable protected area patches excluding medium–high occurrence

10.7717/peerj.1346/supp-7 Supplemental Information 7 Lion occurrence in Namibia (according to Stein et al., 2012) and suitable protected area patches excluding medium–high occurrence

10.7717/peerj.1346/supp-8 Supplemental Information 8 Safety buffer exclusion around registered urban areas (according to Namibia Statistics Agency, 2012)

10.7717/peerj.1346/supp-9 Supplemental Information 9 Potential cheetah recipient areas in Namibia without site fidelity considerations

## Additional Information and Declarations

### Field Study Permissions

The following information was supplied relating to field study approvals (i.e., approving body and any reference numbers): Research activities were endorsed by the Ministry of Environment and Tourism—permit numbers: 1254/2008; 1354/2009; 1459/2010; 1459/2011; 1782/2013; 1888/2014.

### Data Availability

The following information was supplied regarding data availability: These data are currently confidential until two publications are released. At that time, we will deposit all original movement data with the online repository Movebank: https://www.movebank.org/.
