# Supplementary material for: Cheetahs (Acinonyx jubatus) running the gauntlet: an evaluation of translocations into free-range environments in Namibia
Source: PeerJ. 2015 Oct 22;3:e1346. doi: 10.7717/peerj.1346 (PMC4627913; doi:10.7717/peerj.1346)
Supplement: Supplemental Information 1 [file peerj-03-1346-s001.pdf]

Supplemental Information 1 – Additional detail for captivity, immobilisation, transport, and data sharing protocols for translocated cheetahs in Namibia.

Captivity

During intermediate captivity, cheetahs were maintained in enclosures that reflected their natural habitat (*Acacia*-encroached bush savannah) in central Namibia. Captive facilities were in compliance with the national guidelines for the keeping of large carnivores (Ministry of Environment and Tourism, 2012). Enclosure sizes varied between 90 m^2 - 4.3 ha depending on group size, social composition and length of temporary captivity. All enclosures allowed permanent access to water and provided sufficient shade and opportunities to seek cover. Electric fencing (max. 9000 V with low amperage) prevented escapes and direct interaction with wild conspecifics that occurred around the captive facility.

Cheetahs were fed six days per week with horse (*Equus spp.*), donkey (*E. africanus asinus*) or game meat, including warthog (*Phacochoerus africanus*), springbok (*Antidorcas marsupialis*), oryx (*Oryx gazella*), red hartebeest (*Alcelaphus caama*), blue wildebeest (*Connochaetes taurinus*), common impala (*Aepyceros melampus*), plains zebra (*E. quagga*) and greater kudu (*Tragelaphus strepsiceros*). Diets included red meat, bone material, organs, offal and fur provided in aliquots of approximately 2.0 kg per cheetah per feed. Whenever possible, we provided feeds as whole carcasses with subsequent starve days. Although provision of live prey to maintain or train hunting skills (*cf.* Houser et al., 2011) was not permitted (Ministry of Environment and Tourism, 2012), all cheetahs were known to hunt successfully. We observed kills by all long-term captives (including orphans) when hares (*Lepus spp.*),

guineafowl (*Numida meleagris*), or yellow mongoose (*Cynictis penicillata*) naturally entered enclosures.

Generally, cheetahs were housed in their original group compositions, e.g. mothers with dependent offspring, and sibling groups trapped together. Two artificial groups were established from long-term captive orphans captured on different occasions (groups Aju29/30, and Aju42/43/44 in Table 1 respectively). The animals showed no signs of aggression and readily bonded when introduced to each other at approximately 12 months of age. Artificial group formation was undertaken as this was believed to benefit the animals' post-release chances of success and to prevent reproduction by related males and females. To facilitate familiarity, male Aju19 and female Aju20 were introduced to each other during the last week prior to a joint release on the same reserve, but these individuals showed no tendency to bond. None of the attempts to introduce previously unfamiliar cheetahs resulted in hostile interactions or injuries.

Immobilisation

Until October 2010, cheetahs were immobilised with Hellabrunn mixture (Wiesner, 1998), a combination of 100.0 mg/ml Ketamine with 125.0 mg/ml Xylazine at a mixed dose of 0.2 ml per estimated 5 kg body mass. The Xylazine component was reversed with an intramuscular or intravenous administration of Yohimbine at a dose of 0.1 mg/kg body mass. From October 2010 onwards, a combination of Ketamine with Medetomidine was used to induce immobilisation. Cheetahs were injected with 10.0 mg Ketamine (100.0 mg/ml solution) and 0.25 mg Medetomidine (10.0 mg/ml solution) per estimated 5 kg body mass. The Medetomidine component was reversed with 0.50 – 0.75 mg Atipamezole per estimated 5 kg body mass.

During immobilisation we monitored heart rate, body temperature and respiration at least every 5 min. Precautions were taken to avoid zoonotic disease transmission, undue disturbance, and stress to animals. To prevent dehydration and irritation of the cornea, artificial tear fluid was administered and eyes were covered with blindfolds. Capture-related facial abrasions were disinfected and treated with Terramycin wound spray.

Transportation

During transportation in grass-padded, closed/covered crates, cheetahs were not under the influence of immobilising agents. The ventilated crates allowed safe transportation whilst limiting the possibility of injury. We moved female cheetahs with dependent offspring in the same crate.

Data sharing protocol

We projected positional data with Google Earth high-resolution imagery on a regular basis and subsequently distributed information via email. We supplied Google Earth enabled meta-data (from GPS satellite units) to allow land managers to view position data at any required scale and resolution. Emails further included a description of the animal's movements and activities. We emailed information daily during the first months post-release and less regularly when animals settled into definable ranges. We encouraged land managers to share information with other managers and utilise tracking information to adjust their livestock and game husbandry practices, or for non-consumptive tourism activities. We asked managers to investigate suspected cheetah kills after the animal had moved away from the site and frequently received valuable feedback on prey, reproduction and conflict involvement.
