# Supplementary material for: Cheetahs (Acinonyx jubatus) running the gauntlet: an evaluation of translocations into free-range environments in Namibia
Source: PeerJ. 2015 Oct 22;3:e1346. doi: 10.7717/peerj.1346 (PMC4627913; doi:10.7717/peerj.1346)
Supplement: Supplemental Information 2 [file peerj-03-1346-s002.pdf]

Supplemental Information 2 - Classification used to determine degree of habituation of translocated cheetahs.

Degree of habituation	Specific behavioural response
Wild	Subject does not tolerate human presence in any context;<br>Subject exhibits typical natural avoidance behaviour and flight response – no direct observation possible;<br>Subject exhibits strong signs of stress/discomfort when confined – hissing, spitting, stomping, attempt to escape.
Semi-habituated	Except for feeding context, subject always avoids human presence;<br>Subject exhibits expected flight response after food is obtained and seeks cover;<br>Subject exhibits obvious signs of stress/discomfort when confined – hissing, spitting, stomping.
Habituated	Subject tolerates human presence beyond feeding events;<br>Subject does not exhibit typical flight or avoidance behaviour when approached;<br>No direct/physical contact possible (contrary to tame);<br>Subject exhibits reduced signs of stress/discomfort when confined (e.g. only mild irritation).
