# Supplementary material for: Cheetahs (Acinonyx jubatus) running the gauntlet: an evaluation of translocations into free-range environments in Namibia
Source: PeerJ. 2015 Oct 22;3:e1346. doi: 10.7717/peerj.1346 (PMC4627913; doi:10.7717/peerj.1346)
Supplement: Supplemental Information 3 [file peerj-03-1346-s003.pdf]

Supplemental Information 3 – Key characteristics of cheetah recipient reserves.

ID (Region) Location	Size in km^2	Key climate characteristics (Source: Mendelsohn et al., 2002)	Key habitat and vegetation characteristics (Source: Mendelsohn et al., 2002)	Key prey species	Documented predator guild (density – Hanssen & Stander, 2004)	Connectivity with other conservation areas	Animals received
NamibRand Nature Reserve (Hardap)<br>GPS:<br>S -24.97068<br>E 16.02452	1,722	Arid<br>Annual rain: 100-150mm (50-70% variation)<br>Erratic rains<br>Regular droughts	Habitat: Namib plains with prominent inselbergs<br>Biome: Nama-Karoo and Desert<br>Vegetation structure: mixed southern desert and desert – dwarf shrub transition<br>Features:<br>Sand dunes<br>Open grass plains with rock and gravel substrate<br>Dwarf shrub savannah<br>Mountain escarpment<br>Artificial water holes<br>No fencing	steenbok, klipspringer, springbok, oryx, red hartebeest, greater kudu, mountain zebra, plains zebra, blesbok, ostrich	leopard (low-medium), cheetah (low), spotted hyaena (medium-high), brown hyaena (low-medium), black-backed jackal, caracal	Yes	Aju01,02,03<br>Aju07 + 2 cubs<br>Aju17<br>Aju18<br>Aju29,30<br>Aju58 + 2 cubs
Sandfontein Private Nature and Game Reserve (Karas)<br>GPS:<br>S -28.6752<br>E 18.59589	760	Arid<br>Annual rain: 50-100mm (50-60% variation)<br>Erratic rains<br>Regular droughts	Habitat: Gomkab Basin with dissecting rolling hills<br>Biome: Nama Karoo<br>Vegetation structure: Karas dwarf shrubland<br>Features:<br>Open grass plains with rock and gravel substrate<br>Dwarf shrub savannah<br>Inselbergs<br>Mountain escarpment<br>Artificial water holes<br>Access to permanent Orange River<br>Riverine woodland<br>Game fencing	steenbok, common duiker, klipspringer, springbok, greater kudu, oryx, red hartebeest, plains zebra, common eland, common impala, ostrich	leopard (low), cheetah (n/a), spotted hyaena (n/a), brown hyaena (low), black-backed jackal, caracal	No	Aju42,43,44<br>Aju59 + 3 cubs
Kulala Wilderness Reserve (Hardap)<br>GPS:<br>S -24.70415<br>E 15.82466	339	Arid<br>Annual rain: 50-150mm (50-60% variation)<br>Erratic rains<br>Regular droughts	Habitat: Namib plains with prominent inselbergs<br>Biome: Nama-Karoo<br>Vegetation type: desert – dwarf shrub transition<br>Features:<br>Sand dunes<br>Open grass plains with rock and gravel substrate<br>Dwarf shrub savannah<br>Artificial water holes<br>No fencing	steenbok, klipspringer, springbok, oryx, greater kudu, ostrich	leopard (low), cheetah (low), spotted hyaena (medium-high), brown hyaena (low-medium), black-backed jackal, caracal	Yes	Aju19,20
Namib Desert Lodge (Khomas and Hardap)<br>GPS:<br>S -23.92416<br>E 15.81008	250	Arid<br>Annual rain: 100-150mm (50-70% variation)<br>Erratic rains<br>Regular droughts	Habitat: Namib plains with prominent inselbergs<br>Biome: Nama-Karoo<br>Vegetation type: desert – dwarf shrub transition<br>Features:<br>Sand dunes<br>Open grass plains with rock and gravel substrate<br>Dwarf shrub savannah<br>Mountain escarpment<br>Artificial water holes<br>No fencing	steenbok, klipspringer, springbok, oryx, greater kudu, ostrich	leopard (low-medium), cheetah (low), spotted hyaena (medium), brown hyaena (low-medium), black-backed jackal, caracal	Yes	Aju40,41
Frauenstein - Ondekaremba Complex (Khomas)<br>GPS:<br>S -22.40366<br>E 17.42077	112	Semi-arid<br>Annual rain: 350-400mm (30-40% variation)<br>Variable rains<br>Irregular droughts	Habitat: Khomas Hochland Plateau<br>Biome: Savannah<br>Vegetation type: Highland shrubland<br>Features:<br>Undulating terrain<br>*Acacia*-encroached thickets with open glades<br>Artificial water holes<br>Cattle and game fencing	steenbok, common duiker, springbok, oryx, greater kudu, red hartebeest, ostrich, warthog	leopard (medium), cheetah (medium), brown hyaena (low), black-backed jackal, caracal	No	Aju26<br>Aju34<br>Aju56 + 3 cubs<br>Aju65,66
Solitaire Guest Farm (Khomas)<br>GPS:<br>S -23.88794<br>E 16.01535	95	Semi-arid - Arid<br>Annual rain: 100-200mm (50-60% variation)<br>Erratic rains<br>Regular droughts	Habitat: Namib plains with prominent inselbergs<br>Biome: Nama-Karoo<br>Vegetation type: desert – dwarf shrub transition<br>Features:<br>Open grass plains with rock and gravel substrate<br>Dwarf shrub savannah<br>Mountain escarpment<br>Artificial water holes<br>Cattle and game fencing	steenbok, klipspringer, springbok, oryx, greater kudu, ostrich	leopard (low-medium), cheetah (low), spotted hyaena (medium-high), brown hyaena (low-medium), black-backed jackal, caracal	Yes	Aju38
