# Supplementary figures and images for: Cheetahs (Acinonyx jubatus) running the gauntlet: an evaluation of translocations into free-range environments in Namibia
Source: PeerJ. 2015 Oct 22;3:e1346. doi: 10.7717/peerj.1346 (PMC4627913; doi:10.7717/peerj.1346)

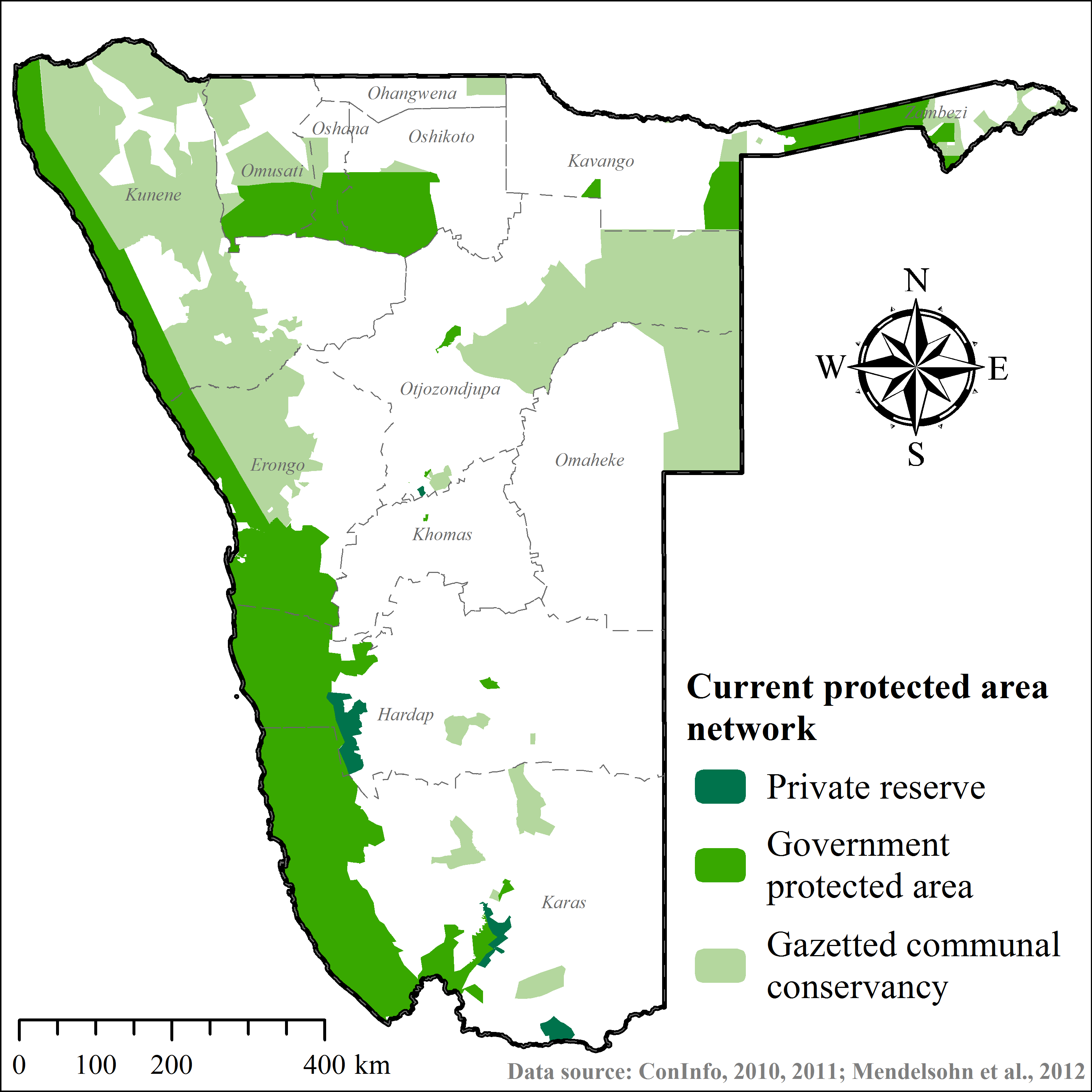

Supplement: Supplemental Information 4 [file peerj-03-1346-s004.png]

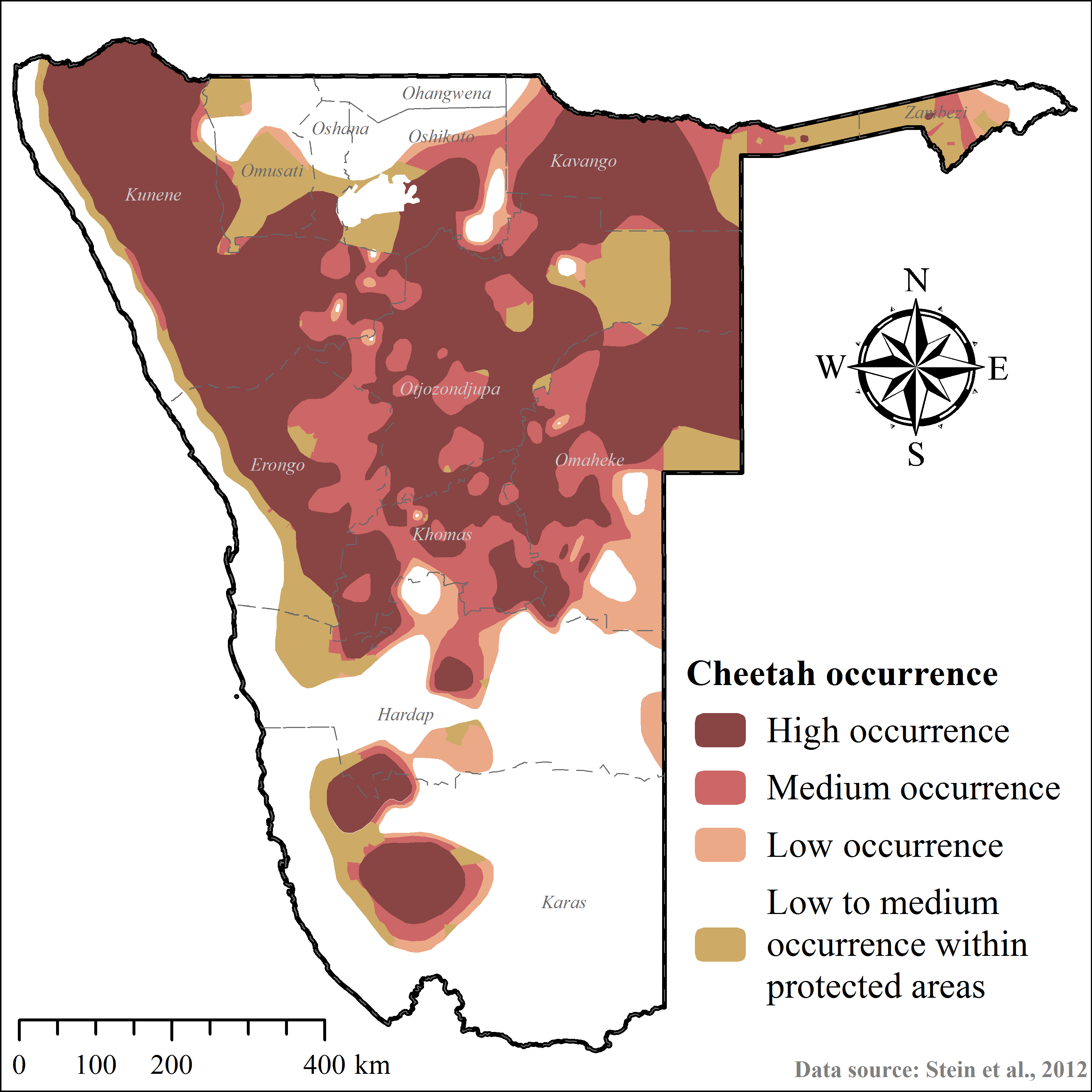

Supplement: Supplemental Information 5 [file peerj-03-1346-s005.png]

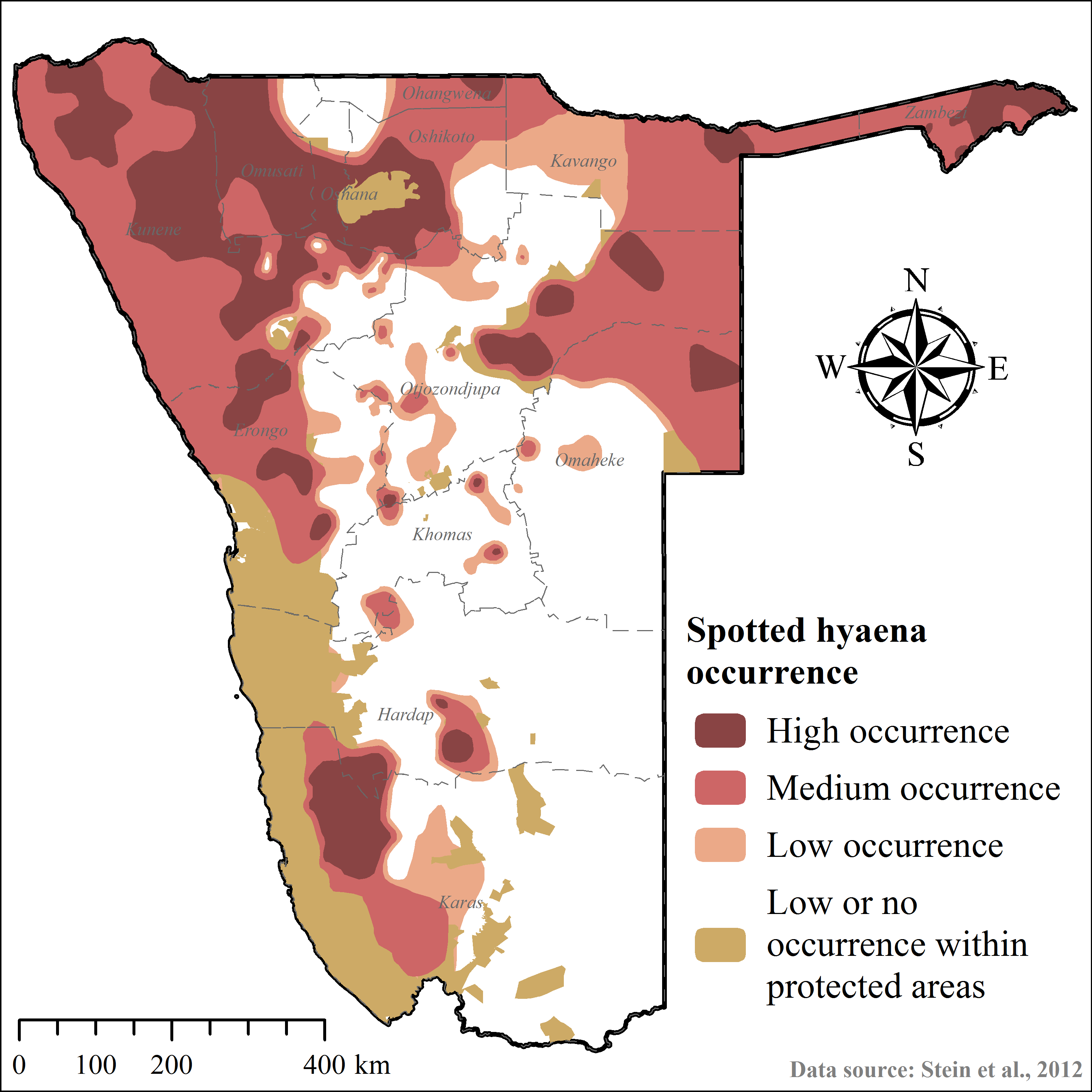

Supplement: Supplemental Information 6 [file peerj-03-1346-s006.png]

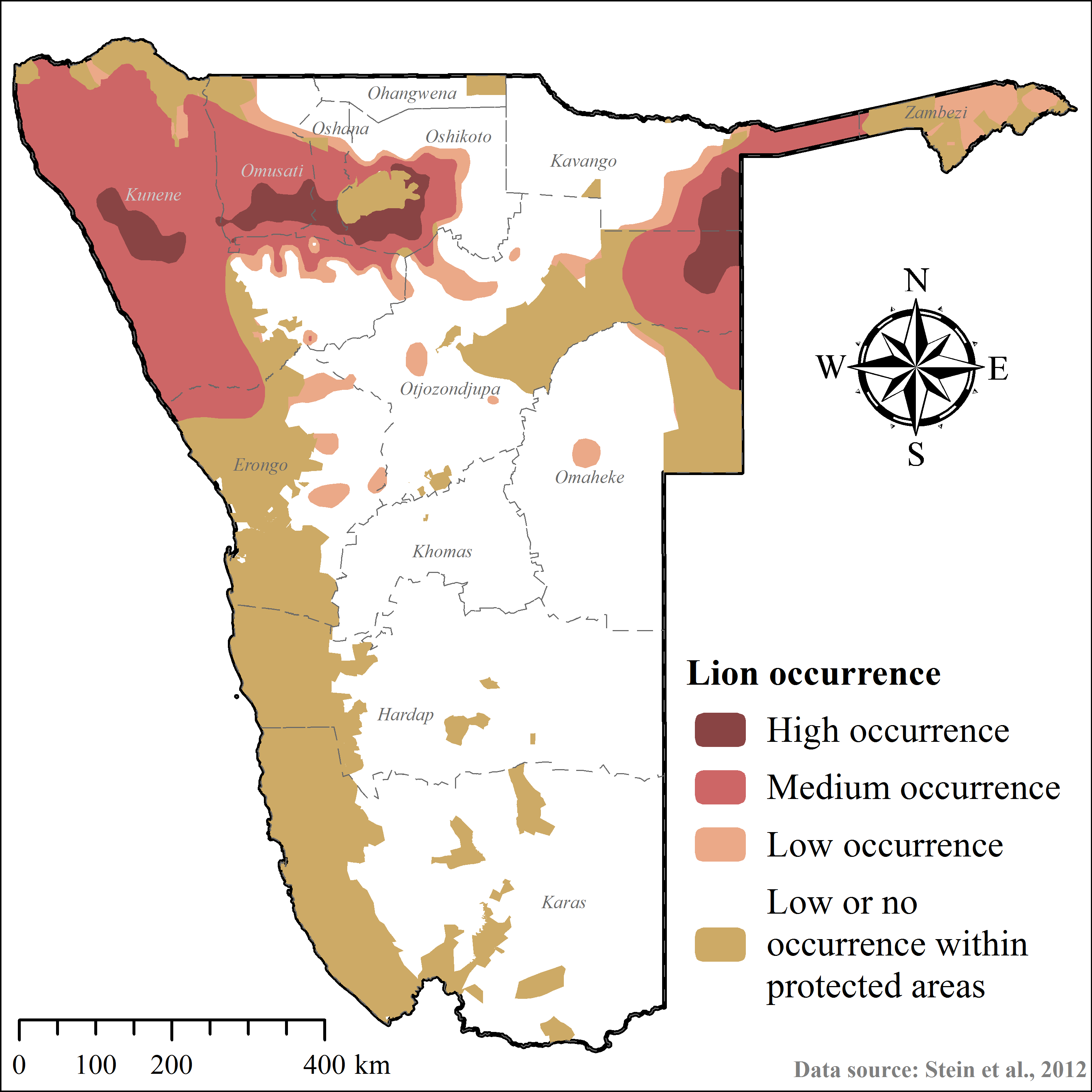

Supplement: Supplemental Information 7 [file peerj-03-1346-s007.png]

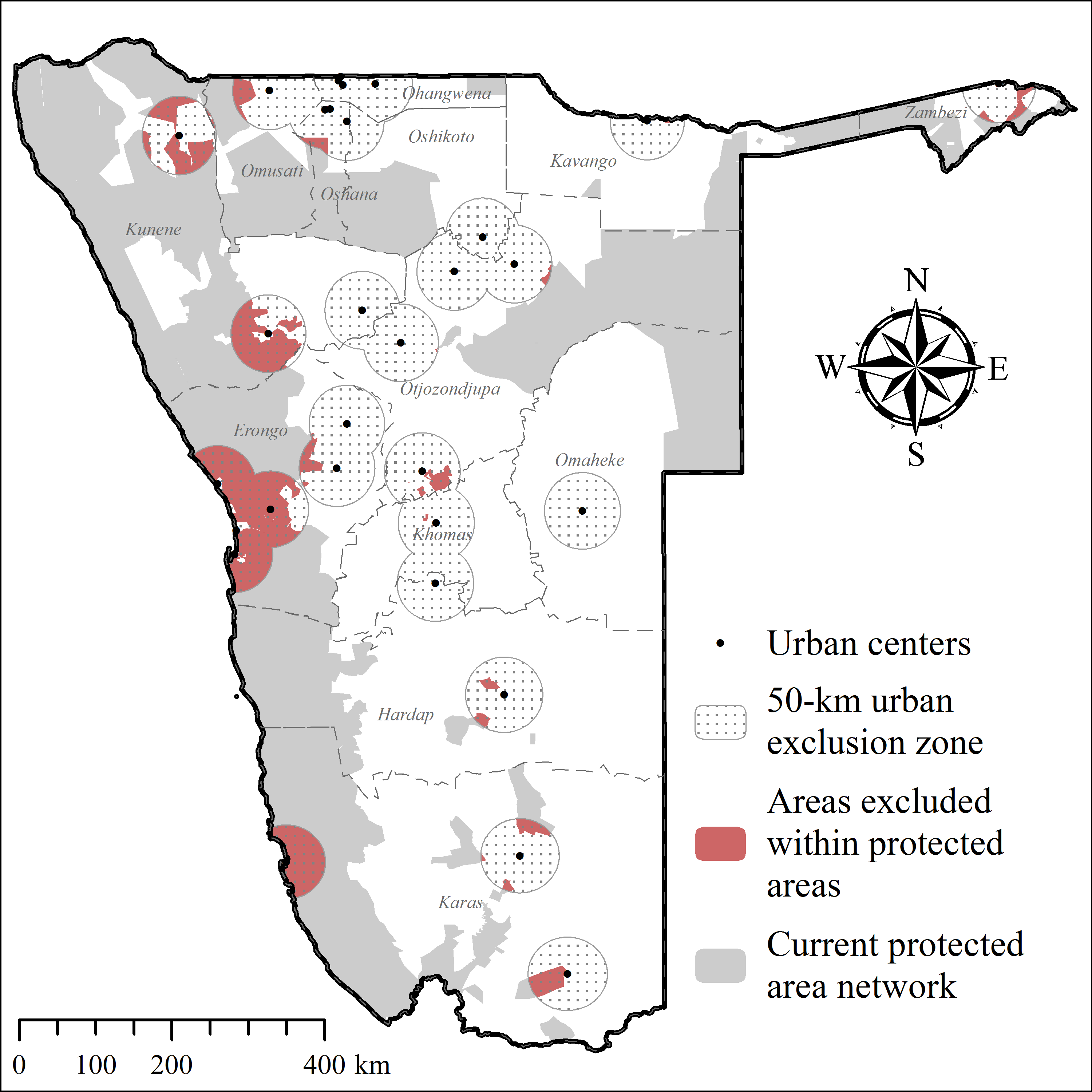

Supplement: Supplemental Information 8 [file peerj-03-1346-s008.png]
