# Supplementary material for: Cheetahs (Acinonyx jubatus) running the gauntlet: an evaluation of translocations into free-range environments in Namibia
Source: PeerJ. 2015 Oct 22;3:e1346. doi: 10.7717/peerj.1346 (PMC4627913; doi:10.7717/peerj.1346)
Supplement: Supplemental Information 9 [file peerj-03-1346-s009.pdf]

Supplemental Information 9 – Potential cheetah recipient areas in Namibia without site fidelity considerations.

ID (region)	Suitable patch size (km^2)	Protected area category
Namib Naukluft Park (Hardap/Erongo)	10,785.3	National park
Otjombinde (Omaheke)	1,738.0	Communal conservancy
Otjituuo 1 (Otjozondjupa)	765.6	Communal conservancy
!Han /Awab (Karas)	672.7	Communal conservancy
Sperrgebiet (Karas)	351.5	National park
Otjituuo 2 (Otjozondjupa)	294.4	Communal conservancy
African Wild Dog (Otjozondjupa)	262.0	Communal conservancy
N=/=a Jaqna (Otjozondjupa)	96.9	Communal conservancy
Ai-Ais Hot Springs (Karas)	54.8	National park
Ozonahi (Otjozondjupa)	50.1	Communal conservancy
